# JRM-28, a Novel HDAC2 Inhibitor, Upregulates Plasticity-Associated Proteins in Hippocampal Neurons and Enhances Morphological Plasticity via Activation of CREB: Implications for Alzheimer’s Disease

**DOI:** 10.3390/cells13231964

**Published:** 2024-11-27

**Authors:** A. F. M. Towheedur Rahman, Sarojini Bulbule, Jawad Bin Belayet, Anna Benko, Carl Gunnar Gottschalk, David N. Frick, Leggy A. Arnold, M. Mahmun Hossain, Avik Roy

**Affiliations:** 1 Department of Chemistry and Biochemistry, University of Wisconsin-Milwaukee, 2000 E Kenwood Blvd, Milwaukee, WI 53211, USA.; rahman25@uwm.edu (A.F.M.T.R.); sb@simmaron.com (S.B.); jbealyet16@gmail.com (J.B.B.); abenko@uwm.edu (A.B.); ggottschalk@simmaron.com (C.G.G.); frickd@uwm.edu (D.N.F.); arnold2@uwm.edu (L.A.A.); 2 Simmaron Research Institute, 948 Incline Way, Incline Village, NV 89451, USA; 3 Milwaukee Institute for Drug Discovery, 2000 E Kenwood Blvd, Milwaukee, WI 53211, USA; 4 Simmaron Research and Development Laboratory, University of Wisconsin-Milwaukee, Chemistry Building, 2000 E Kenwood Blvd, Suite # 320, Milwaukee, WI 53211, USA

**Keywords:** Histone deacetylase 2, cAMP responsive element binding protein 2, NMDA receptor subunit 2A, AMPA-sensitive glutamate receptor 1, immunofluorescence, immunoblot, HDAC inhibitor, triethyl amine, dichloromethane, benzene, lithium diisopropylamide, tetrahydrofuran, iodine, sodium acetate, methanol, meta-chloroperoxybenzoic acid

## Abstract

Enhancement of neuronal plasticity by small-molecule therapeutics protects cognitive skills and also ameliorates progressive neurodegenerative pathologies like Alzheimer’s disease (AD) and dementia. One such compound, a novel histone deacetylase 2 (HDAC2) inhibitor named JRM-28, was shown here to enhance dendritic strength, augment spine density, and upregulate post-synaptic neurotransmission in hippocampal neurons. The molecular basis for this effect correlates with JRM-28-induced upregulation of the transcription of cAMP response element-binding protein(CREB), induction of its transcriptional activity, and subsequent stimulation of expressions of CREB-dependent plasticity-associated genes, such as those encoding N-methyl-D-aspartate (NMDA) receptor subunit NR2A and the α-amino-3-hydroxy-5-methyl-4-isoxazolepropionic acid (AMPA) receptor subunit GluR1. Specifically, JRM-28 stimulated the NMDA- and AMPA-receptor-sensitive ionotropic calcium influx in hippocampal neurons. Interestingly, JRM-28 did not induce NMDA- and AMPA-sensitive calcium influx in hippocampal neurons once the expression of CREB was knocked down by *creb* siRNA, suggesting the critical role of CREB in JRM-28-mediated upregulation of synaptic plasticity. Finally, JRM-28 upregulated CREB mRNA, CREB-dependent plasticity-associated markers, and ionotropic calcium influx in iPSC-derived AD human neurons, indicating its therapeutic implications in the amelioration of AD pathologies.

## 1. Introduction

Morphological plasticity [1] is a discipline of neuroscience that evaluates the roles of structural components of neurons such as dendritic arborization [2], spine density [3], spine maturation [4], and post-synaptic neurotransmission [5] in the regulation of neuronal function [6]. Studies have shown that the upregulation of dendritic spine density [7], augmentation of spine maturation [8], and enhancement of dendritic strength [9] support neurodevelopmental cognition and also play a pivotal role in the experience-based remodeling of neurons in early adulthood [10]. Apart from these physiological effects, morphological plasticity renders cognitive resilience against Alzheimer’s disease (AD) [11,12], a devastating neurodegenerative disorder that severely impairs cognitive functions such as learning and memory. Therefore, the induction of morphological plasticity could be critical both in the amelioration of AD pathogenesis and in the preservation of cognitive function in a healthy brain.

Histone deacetylase 2 (HDAC2), a class-I histone deacetylase enzyme [13], represses gene expression by catalyzing the deacetylation of histones. By this mechanism, HDAC2 has been reported to suppress expressions of many neuroprotective molecules [14], including brain-derived neurotrophic factor (BDNF) [15] and the cellular homolog of v-FOS (c-FOS) proteins [16]. Moreover, similar to HDAC6 [17], the activation of HDAC2 was also shown to impair morphological plasticity via destabilization of cytoskeletal protein α-tubulin by removing an acetate from lysine 40 of α-tubulin [18]. As a result, the overexpression of HDAC2 was shown to reduce the number of synapses in hippocampal neurons, causing impaired hippocampal learning and memory formation in mice [17,18]. Therefore, pharmacological inhibition of HDAC2 could have a strong implication in augmenting morphological plasticity and also in preserving cognitive function in the AD brain. 

Structurally, macrocyclic HDAC inhibitors (HDACi) [19] share common structural determinants, including a cap group, a linker, and a zinc-binding group (ZBG). The crystal structures of HDACi-Zn^2+^ complexes demonstrated that the zinc-binding group chelates with a zinc ion (Zn^2+^) located in the catalytic center of the HDAC enzyme [20]. The linker is located in a narrow hydrophobic channel (11 Å) connecting the ZBG with the surface recognition cap group [21]. Modifying these distinct parts of HDACi is critical for improving their potency and isoform selectivity. Previously, a novel HDACi termed MJM-1 was developed to induce hippocampal learning and memory [22]. MJM-1 inhibited HDAC2 with an IC_50_ value of 10 µM. The inhibition of other HDAC isoforms by MJM-1 warranted the development of more selective and potent HDAC2 inhibitors.

We report the design, synthesis, and evaluation of a novel HDACi named JRM-28. Immunofluorescence (IF) analyses combined with quantification studies revealed that 5 µM JRM-28 upregulated the cellular expressions of dendritic markers such as MAP2, NR2A, and gluR1 and promoted the maturation of dendritic spines in mouse primary hippocampal neurons. While exploring the molecular mechanism, different immunological analyses and a *cre*-luciferase assay indicated that by suppressing HDAC2 activity, JRM-28 upregulated the expression and transcriptional activity of CREB not only in mouse primary neurons but also in iPSC-derived human AD neurons. Collectively, our current study highlights the discovery of a novel HDAC2i JRM-28 that augments the morphological plasticity in hippocampal neurons by inducing transcriptional activation of CREB.

## 2. Materials and Methods

### 2.1. Reagents and Cell Lines

SHSY5Y, an immortalized neuronal cell line, was purchased from ATCC (Cat# CRL-2266). A 31-year-old female AD patient (A246E mutation at *psen1* gene)-derived neural stem cells (NSCs) were purchased from AXOL Bioscience Ltd. (Cat# ax0114, Midlothian, UK). Regular cell culture supplies, including neurobasal medium (Cat# 21103049; Gibco^TM^, NY), B-27™ Plus Supplement (Cat# A3582801; Gibco^TM^, NY), serum-free B-27™ supplement (Cat# 17504001; Gibco^TM^, NY), 0.05% trypsin-EDTA (Cat# 25300062), Dulbecco’s Modified Eagle Medium (DMEM)/F-12 (Cat# 11320033; Gibco^TM^, NY), heat-inactivate fetal bovine serum (FBS) (Cat# 16140071; Gibco^TM^, NY), L-glutamine (Cat# 21051024), antibiotic-antimycotic solutions (Cat# 15240096), and Nalgene™ general long-term storage cryogenic tubes (Cat# 5000-0020) were purchased from Thermo Fisher Scientific (Waltham, MA, USA). Specialized cell culture reagents for iPSC-derived neural stem cell culture, including KnockOut^TM^ DMEM/F12 media (Cat# 12660012), StemPro^TM^ Neural Supplement (Cat# A10508-01), recombinant human FGF-basic (AA10-155) protein (Cat# PHG0024), recombinant human EGF (Cat# PHG0314), GlutMAX^TM^ Supplement 200 mM (Cat# 35050061), StemPro^TM^ Accutase (Cat# A1105), and Geltrex^TM^ basement membrane matrix (Cat# A14133), were purchased from ThermoFisher Scientific. Western blot supplies, including 0.45 μm nitrocellulose membrane (Cat# LC2001; Thermo Fisher Scientific), Novex™ Value™ 4 to 12% tris-glycine, 1.0 mm mini protein gel (Cat# XV04120PK20; Thermo Fisher Scientific), TBS-blocking buffer (Cat#; Licor Biosciences, Lincoln, NE, USA), IRDye 680-conjugated secondary antibody (Cat#; Licor Biosciences), IRDye800-conjugated secondary antibody (Cat#; Licor Biosciences), and Chameleon^®^ duo pre-stained protein ladder (Cat# 928-60000; Licor Biosciences), were purchased from various vendors. For calcium entry assay, Fluo-4 Direct™ Calcium Assay Kit (Cat# F10471), 96-well fluorescence microplates (Cat# M33089), N-methyl-D-aspartic acid (Cat# J61361.MF), and (+/−)-alpha-amino-3-hydroxy-5-methyl-4-isoxazolepropionic acid (Cat# J64884.MA) were purchased from Thermo Fisher Scientific. For transfection assay, Lipofectamine™ 3000 transfection reagent (Cat# L3000015) and pre-designed/validated creb siRNA (Cat# AM16708) were purchased from Thermo Fisher Scientific, whereas CRE dual luciferase (Firefly/Renilla) was purchased from BPS Biosciences (Cat# 60611). For the immunofluorescence (IF) assay, FITC-conjugate goat anti-mouse (Cat# A16085) 2° antibody, TRITC-conjugated anti-rabbit (Cat# A16101) 2° antibody, CY5-conjugated Phalloidin (Cat# A30107), and DAPI (Cat# D3571) were purchased from Thermo Fisher Scientific. Cy5-conjugated anti-mouse (Cat# A10524) 2° antibody was purchased from Jackson Laboratories. Information, application, and dilution of all primary and secondary antibodies are summarized in a separate table. For cytotoxicity assay, CyQUANT™ LDH cytotoxicity assay kit (Cat# C20301) was purchased from Thermo Fisher Scientific, and DeadEnd™ Colorimetric TUNEL kit (Cat# G7360) was purchased from Promega (Madison, WI, USA).

### 2.2. Reagents for Chemical Synthesis

For the overall synthesis, triethylamine (Cat# T0886) *n*-butyllithium solution, 2.5 M in hexanes (Cat# 230707), diisopropylamine (Cat# 471224), sodium acetate (Cat# S8750-250G), N-methyl acetanilide (Cat# S392146-50MG), 3-Chloroperbenzoic acid (Cat# 273031-25G), dichloromethane (Cat# 270997-18L-P1), and sodium thiosulfate (Cat# 217263-1KG) were purchased from Millipore Sigma. Triphenylphosphoranylidene acetaldehyde (Cat# AC307491000) and triphenylmethyl mercaptan (Cat# AC140411000) were acquired from Acros. Acrolein (Cat# 103755-5g), ammonium chloride (Cat# S8750-250G), reagent grade hexane (Cat# 102187-200L), and ethyl acetate (Cat# 18-602-510) were purchased from Oakwood Chemicals. Iodine (Cat# AC38705-1000) and acetone (Cat# BDH1101-19L) were purchased from VWR Chemicals (Solon, OH, USA).

### 2.3. Preparation of Structures for Docking

Structures were built in ChemDraw Professional-16 (PerkinElmer, Shelton, CT, USA) and saved as .mol format. To eliminate internal strains originating from the unfavorable bond length, bond angle, and torsion, energy minimization was performed using MOE2022.02 (Molecular Operating Environment, Chemical Computing Group, 910-1010 Sherbrooke W., Montreal, QC H3A 2R7, Canada). First, the .mol file of the desired structure was imported to the MOE interface. Then, from the “Compute” option, the “Energy Minimize” interface was opened. The “MMFF94x” forcefield was selected for molecular mechanics calculation for energy minimization. The gradient parameter was changed from 0.1 to 0.0001 to control the level of minimization accuracy. Finally, the energy minimization algorithm was run to achieve a more realistic 3D structure for docking. 

### 2.4. In Silico Docking of JRM-28a in HDAC2 and HDAC4

Crystal structures of recombinant human HDAC2 (PDB ID: 4lxz) and HDAC4 (PDB ID: 4cbt) enzymes were obtained from the RCSB Protein Data Bank (https://www.rcsb.org/, accessed on 14 December 2021 for HDAC2 and 7 February 2023 for HDAC4). Energy minimization of these structures was performed by the conjugate gradient and steepest descent algorithm to exclude inappropriate contacts of protein atoms. GROMOS 96 43B1 parameters set was used to execute the computations in vacuo. The overall protein preparation for docking was implemented using the Swiss-PDB Viewer Version 4.1.0 (https://spdbv.unil.ch/, accessed on 7 February 2023). The SDF (structure-data format) structure of compound JRM-28 for docking was generated by ChemDraw Professional (Version 16.0, PerkinElmer Informatics, Inc., 710 Bridgeport Avenue, Shelton, CT, USA). The docking at the active site of enzymes was performed by the Molecular Operating Environment (MOE, Version: 2020.0901, Chemical Computing Group Inc., Canada) under the MMFF94x forcefield. The active sites of 4lxz and 4cbt were identified by the Alpha SiteFinder function in MOE software (version 2024) and the coordinates of the ligands at the crystal structure. The active sites were protonated and solvated before the rigid docking simulation was executed. The best five docking poses were generated based on the lowest binding energy, designated by the S-score of the MOE software.

### 2.5. HDAC2 Inhibition Assay

HDAC2 inhibition assay was performed by either the luminometric or fluorimetric method applying the following procedures. The table (Table 1) represents the details of different HDAC proteins and antibodies used in all biochemical assays.

### 2.6. Luminometric Method

HDAC2 inhibition assay was performed by HDAC-Glo™ I/II Assays and Screening System (Catalog# G6420, Promega Corporation, Madison, WI, USA) by the luminometric method according to the manufacturer’s protocol. Human recombinant HDAC-2 (Cat# 50002) was purchased from BPS Bioscience (San Diego, CA 92121, USA). The HDAC-2 inhibition assay was performed as previously described [22] to determine IC values. In brief, the purified HDAC2 was diluted in HDAC assay buffer (provided with the HDAC-Glo™ I/II Assays and Screening System) to the same concentration based on their calculated molar mass. JRM-28 was first activated by reducing with dithiothreitol (DTT) and then serially diluted in HDAC-Glo buffer. Next, 10 µL of JRM-28 after serial dilution was added to 10 µL of diluted HDAC2 in such a way that the final HDAC2 concentration was 0.5 nM in each well of a 384-well microplate (Greiner, Kremsmünster, Austria). After 30 min of incubation, 20 µL of the HDAC-Glo substrate/developing reagent was added to each well according to the manufacturer’s instructions. The 384-well plate was then agitated on an orbital shaker for the mixing of components in each well for another 30 min at room temperature. Finally, luminescence was measured using a POLARstar Omega plate reader (BMG Labtech, Ortenberg, Germany). Luminescence values for each well were fitted using GraphPad Prism (v6) to estimate the concentration of each HDACi needed to inhibit the reaction by 50% (IC_50_), as described earlier [22]. 

### 2.7. Fluorimetric Method

The inhibition of HDAC1, 2, 3, and 6 by JRM-28 was fluorometrically measured using the HDAC Fluorogenic Assay Kit (Catalog# 50034) by BPS bioscience. Purified recombinant human HDAC1 (Cat# 50051), HDAC3 (Cat# 50003), and HDAC6 (Cat# 50056) were also obtained from BPS Bioscience. The enzyme inhibition assay was performed according to the manufacturer’s protocol. Briefly, the test inhibitor solution in DMSO was made in such a way that the final DMSO concentration did not exceed 1%. Purified recombinant HDAC2 (supplied with the fluorogenic kit) was diluted to 6 ng/µL (5 µL/well with the supplied HDAC assay buffer). HDAC 1, 3, and 6 were also diluted to the optimized concentration (5 µL/well) with the HDAC assay buffer. The activity of JRM-28 was measured along with DTT control and DMSO as blank. After adding 5 µL of the diluted HDAC enzymes in designated wells, the 96-well plate (supplied with the kit) was incubated at 37 °C. Then, the reaction was initiated by adding 5 µL of diluted HDAC substrate to each well, followed by another incubation at 37 °C for 30 min. Next, 50 µL of the developer reagent was added to each well and incubated at room temperature for another 15 min. Finally, fluorescence was measured by a microplate absorbance spectrophotometer (PerkinElmer Enspire 2300 Multilabel Multimode Microplate Reader) with an excitation at λ = 485 nm and detection at λ = 528 nm. 

### 2.8. Cell Culture Strategies of Primary Hippocampal Neurons, SHSY5Y, and AD Neurons

Primary neuronal cell culture was performed based on a protocol approved by the Institutional Animal Care and Use Committee (IACUC) of the University of Wisconsin-Milwaukee, Milwaukee, WI-53211, and by the guidelines set by the National Institute of Health. Embryonic day 18 (E18) fetuses were harvested, and hippocampal tissue was dissected from brains and then processed for single cell culture in poly-D-lysine-coated plates, as discussed elsewhere [6,23]. Neuronal cells were maintained with B-27^TM^-plus-supplemented complete neurobasal media for 7–10 days until fully differentiated. After that, the treatment with JRM-28 was performed. SHSY5Y cells were grown, maintained, and passaged with complete DMEM media (a bottle of DMEM supplemented with 10% FBS, 2 mM L-glutamine, and 100 units/mL antibiotics); otherwise, for neuronal differentiation study, SHSY5Y was seeded with complete neurobasal medium. For making AD neurons, commercially purchased (Axol Bioscience Ltd.) A246E *psen1* neural stem cells (NSCs) were maintained with KnockOut^TM^ DMEM/F12 media supplemented with StemPro^TM^ Neural Supplement, bFGF, EGF, 2 mM GlutMAX^TM^ supplement, and 10 mL/L antibiotic-antimycotic solution. These pluripotent NSCs were grown in a 25 mm cell culture flask coated with Geltrex^TM^ matrix until fully confluent and then detached by Accutase, centrifuged, washed, and plated in a poly-D-lysine-coated plate supplemented with complete neurobasal media for neuronal differentiation. After one week, when neurons were differentiated, JRM-28 treatment was conducted.

### 2.9. Lactate Dehydrogenase Assay

Lactate dehydrogenase (LDH) cell cytotoxicity assay was performed per the manufacturer’s protocol. Briefly, SHSY5Y cells were grown in a 96-well plate with up to 70% confluency followed by treatment with increasing doses of JRM-28 under serum-free conditions. Serially diluted JRM-28 was used in a concentration range from 75 nM to 50 μM. As a control, serially diluted DMSO controls were added. After 24 h, supernatants were harvested by a multi-channel pipette and then assessed for LDH release with CyQUANT™ LDH Cytotoxicity Assay Kit. Briefly, 50 μL of supernatant was incubated with an equal volume of the working reaction mixture (generated after combining the 600 µL of assay buffer Stock Solution with the 11.4 mL of Substrate Stock substrate mix), incubated at room temperature for 30 min protected from light, terminated by 50 μL of stop solution, and then measured at 490 nm using a microplate absorbance spectrophotometer (Perkin Elmer Enspire 2300 Multilabel Multimode Microplate Reader). The percentage of cytotoxicity was determined following the formula provided with the LDH assay kit and then converted to relative cell cytotoxicity.

### 2.10. TUNEL Assay

To assess the effect of JRM-28 on apoptosis in SHS5Y cells, a total of 3 × 10^5^ cells suspended in 3.5 mL of complete neurobasal medium was distributed in each well of a poly-D-lysine-coated eight-well Chamber Slide. After 48 h, cells were treated with 2, 5, 10, 20, 30, and 50 μM of JRM-28 under serum-free conditions and then incubated for an additional 24 h. The TUNEL activity was measured by colorimetric microscopy as directed in the protocol described in DeadEnd™ Colorimetric TUNEL System (Promega, Madison, WI, USA). Hematoxylin staining was adopted to counterstain the slide.

### 2.11. Measurement of Spine Density and Size

For counting spine density, dissociated E18 hippocampal neurons maintained 18 days in vitro were stained with Alexa-647 conjugated phalloidin (Cat #A22287) together with MAP2. Only densely stained neurons (>20 spines every 10 µm dendrite) were counted. The total length of each dendrite was measured at 400× magnification using an Accu-Scope EXC-500 microscope (NY, USA). The number of spines on the dendrites was counted under oil immersion. As some of the spines were hidden under the dendrite, only spines that protruded laterally from the shafts of the dendrites into the surrounding area of clear neuropil were counted. The spine density of a pyramidal neuron was calculated by dividing the total number of spines on a neuron by the total length of its dendrites and was expressed as the number of spines/10 µm dendrite. The size of the dendritic spines was measured by calculating the ratio of mean fluorescent intensity (MFI) of the spine head and MFI of the dendritic shaft as described elsewhere [6]. 

### 2.12. Calcium Influx Assay in Neuronal Stem Cells

Mouse primary hippocampal neurons and AD neurons were loaded with Fluo4-fluorescence conjugated calcium dye (Invitrogen Molecular Probes, Cat #F10471) and incubated at 37 °C for 60 min, as described before [23]. Briefly, fluorescence excitation and emission spectra were recorded in a Perkin–Elmer Victor X2 Fluorimetric Spectrometer (Versiti Blood Center, Wauwatosa, WI, USA) in the presence of 50 μM NMDA and AMPA solutions. The recording was performed with 100 repeats at 0.1 ms intervals. The oscillogram was generated with the help of Parkin Elmer 2030 software that was connected to the Victor X2 calcium recorder unit. The real-time calcium noise graph was generated as a kinetic chart plotted on a linear scale.

### 2.13. Semi-Quantitative RT-PCR

Total RNA was isolated from SHSY5Y neurons using Gene JET RNA Purification Kit (Thermofisher Scientific; Cat #K0731) following the manufacturer’s protocol. RNA was converted to cDNA using the GoScript™ Reverse Transcriptase kit (Promega, Cat # A5001). Semi-quantitative RT-PCR was carried out as described earlier [23] using an RT-PCR kit from Promega (Cat# M7502). Briefly, 1 µg of total RNA was reverse-transcribed using oligo(dT)12–18 as a primer and MMLV reverse transcriptase in a 20 µL reaction mixture. The resulting cDNA was diluted by nuclease-free H_2_O in a ratio of 1:2, and diluted cDNA was amplified using the following primers (Table 2).

### 2.14. Immunofluorescence Analysis

Immunofluorescence analysis was performed as described earlier [23,24,25]. Briefly, cells cultured on coverslip contained in six-well plates (Lab-Tek II) were fixed with 4% paraformaldehyde for 20 min followed by two rinses in TBST. Samples were incubated in a 1:1 solution of TBST and blocking buffer containing rabbit anti-NR2A (1:250), anti-GluR1 (1:250), ant-HDAC2 (1:250), anti-CREB (1:250), and anti-MAP2 (1:250) for 2 h at 37 °C. After three washes in PBST (15 min each), slides were further incubated with FIT-C and TRIT-C-conjugated secondary antibodies. For negative controls, a set of culture slides was incubated under similar conditions with secondary antibodies and without the primary antibodies (Table 3). The samples were mounted and observed under an Accuscope fluorescent microscope. The images were captured and merged in CaptaVision^TM^ imaging software.

### 2.15. Immunoblot Assay

Immunoblot analysis was carried out as described earlier [26]. Briefly, SHSY5Y neuronal cell homogenates were electrophoresed by 4–12% Tris-Glycine gradient gel, proteins were transferred onto a 0.45 μm nitrocellulose membrane, and the protein band was visualized with Odyssey Sa infrared scanner after immunolabeling with primary antibodies followed by infrared fluorophore-tagged secondary antibody (Licor Biosciences, Lincoln, NE, USA).

### 2.16. Creb siRNA Transfection

SHSY5Y neurons were transfected with Lipofectamine PLUS^®^ (Invitrogen) and Nupherin-neuron (Biomol, Hamburg, Germany), as described earlier [27]. Briefly, SHSY5Y cells were maintained with transfection media followed by the addition of 0.25 μg (each well in a 6-well plate) or 0.1 µg (each well in a 96-well plate) of creb siRNA complexed with Neupherin peptide and Lipofectamine PLUS^®^. After 5 h, the cells were supplemented with 20% FBS-containing media, and after another 18 h, cells were treated with JRM-28.

### 2.17. CRE Luciferase Assay

CRE luciferase assay was performed using a dual luciferase kit (Cat# 60611; BPS Bioscience). The CRE reporter is premixed with a constitutively expressing Renilla (sea pansy) luciferase vector that serves as an internal control for transfection efficiency. The reporter construct is transfected in SHSY5Y cells with Lipofectamine3000^TM^ transfection reagent mixed with serum-free Opti-MEM^TM^ Transfection media followed by supplementation with 20% FBS-containing media. After 24 h, cells were treated with JRM-28 for 5 h, and then dual luciferase activity was measured by cell lysis. The reaction was set in a 96-well plate, and the assay endpoint was recorded in a PerkinElmer Enspire multimodal plate reader.

### 2.18. Statistical Analyses

All statistical analyses were performed in GraphPad Prism 9. An unpaired t-test was performed when comparing means between the two groups. One-way ANOVA was performed when analyzing the significance of the means in multiple groups with a single effector of treatment. The null hypothesis is rejected at *p* < 0.05, analysis with treatment as an effector. For correlation analyses, the first D’Agostino–Pearson normality test was performed. If the dataset passed the normality test, then a parametric Pearson correlation analysis was performed; otherwise, a non-parametric Spearman correlation statistic was performed. Results are plotted as mean ± SD of three different experiments.

## 3. Results

### 3.1. Synthesis and Characterization of Novel HDAC2 Inhibitor JRM-28

Inhibition of HDAC2 is beneficial in the amelioration of neurodegenerative pathologies in Alzheimer’s disease (AD) [28], Parkinson’s disease (PD) [29], and ischemic stroke [30]. Therefore, the application of selective HDAC2 inhibitors could have strong therapeutic implications in the treatment of neurodegenerative diseases. However, due to the remarkable structural similarity of all class-I HDACs (HDAC1/2/3 and 8), a major drawback of currently available HDAC2 inhibitors is the limited specificity, as these pharmacophores target all class-I HDACs with variable efficiency [31].

**Figure 1:**
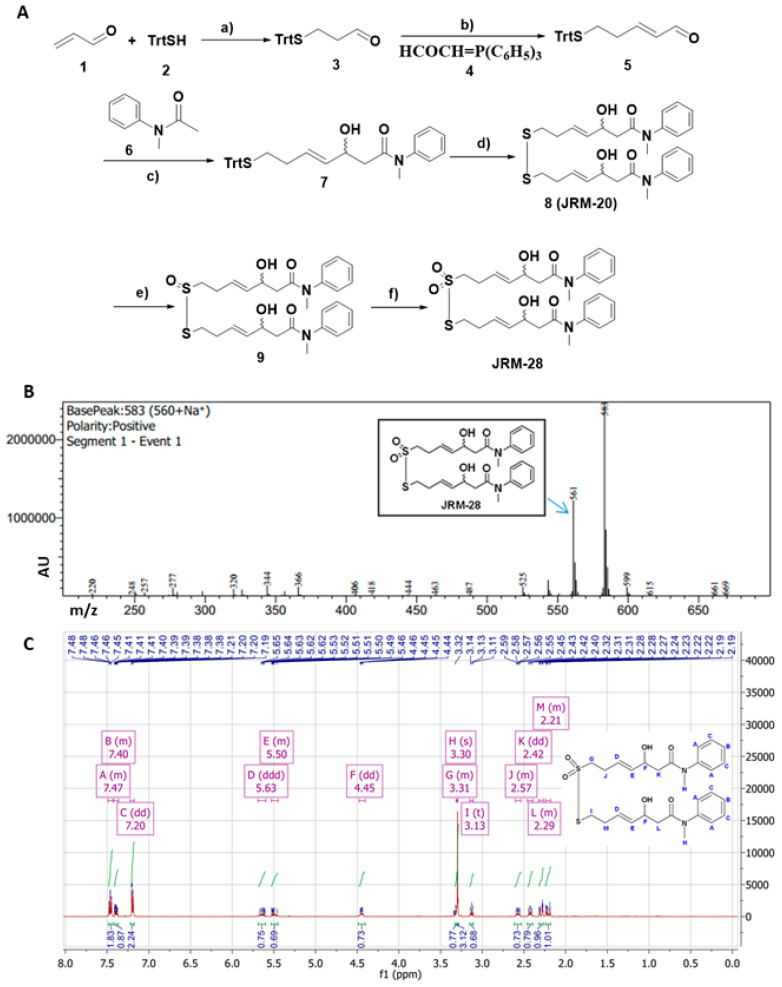
Synthesis and characterization of JRM-28. (**A**) A schema for JRM-28 synthesis. Reagents and conditions: (a) Et_3_N, CH_2_Cl_2_, r.t., 3 h; (b) 2-(triphenylphosphoranylidene) acetaldehyde, C_6_H_6_, reflux, 12 h; (c) LDA, THF, −78 °C; (d) I_2_, NaOAc, CH_2_Cl_2_: MeOH (10:1), 0 °C to r.t., 3 h; (e) mCPBA, CH_2_Cl_2_, reflux 40 °C, 12 h; (f) mCPBA, CH_2_Cl_2_, r.t. to 40 °C, reflux, 2 h; (**B**) Mass spectra of JRM-28 in positive ion mode with the base peak at 561 (C_28_H_36_ N_2_O_6_S_2_+H^+^) and (**C**) ^1^H-NMR of JRM-28 with peak assignment confirm the successful synthesis of the proposed compound (details discussed in Supplementary Methods section). A chemical shift value was recorded for each equivalent proton (marked with alphabet from A to M) and shown as ppm unit within enclosures in the NMR spectra. (Acronyms: Et_3_N = triethyl amine, CH_2_Cl_2_ = dichloromethane, C_6_H_6_ = benzene, LDA = lithium di-isopropylamide, THF = tetrahydrofuran, I_2_ = iodine, NaOAc = sodium acetate, MeOH = methanol, mCPBA = meta-chloroperoxybenzoic acid).

Based on previous structure-activity relationship studies [22], we designed and synthesized JRM-28. See Supporting Information for a detailed description of synthetic methods and product characterizations. Briefly, JRM-28 was synthesized by reacting triphenylmethyl mercaptan with acrolein via a conjugate addition reaction followed by a Witting reaction to afford compound **5** (Figure 1A). N-methyl acetanilide was employed as a lithium enolate to obtain the corresponding benzamide monomer **7**. The monomer was then dimerized in the presence of iodine and sodium acetate. The dimer **8** was successively oxidized twice at C-7 of disulfide linker with meta-chloroperoxybenzoic acid (mCPBA) to afford the final product, JRM-28. The mass of JRM-28 was confirmed by mass spectrometry, showing 561 *m/z* for the M+1 product and 583 *m/z* for the M + Na^+^ species (Figure 1B). The structure of JRM-28 was confirmed by ^1^H NMR (Figure 1C). The step-by-step synthesis is explained in detail in the Supplementary Methods, and NMR validations of each intermediate are displayed in Supplementary Figures S1–S15.

The dose-dependent inhibition of HDAC2 by JRM-28 was measured by luminescence using recombinantly expressed protein. The assay was performed in the presence and absence of dithiothreitol (DTT), a thiol-containing reducing agent. As depicted previously [32,33], romidepsin, a potent HDAC inhibitor, displayed a strong HDAC inhibitory effect once its disulfide bond was reduced with DTT [34,35]. Since JRM-28 is a dimer with a similar disulfide linkage, we proposed the reduction of JRM-28 by DTT, forming two different monomers. The monomer with the sulfinic acid (SO_2_H) moiety was termed JRM-28a, and the monomer with the sulfhydryl (SH) group was termed JRM-28b. The justification of DTT treatment and relevant schema (Supplementary Figure S16) are included in the Supplementary Materials.

**Figure 2:**
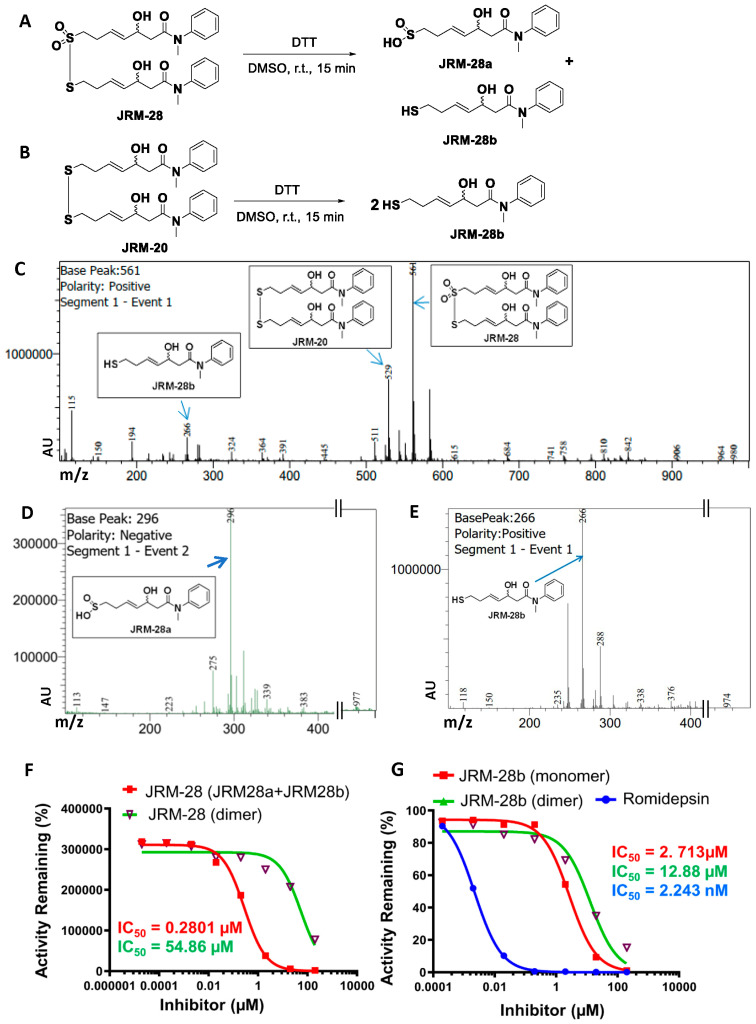
HDAC2 inhibitory role of JRM-28 and its monomers JRM-28a and b. Schematic presentations of the reduction of JRM-28 to JRM-28a and JRM-28b (**A**) and the reduction of JRM-20 to JRM-28b (**B**) by DTT. Before the HDAC2 inhibition assay, a 10 mM solution of both JRM-28 and its precursor, JRM-20, in DMSO was reduced by 350 mM aqueous DTT solution. The mass spectrum of the sample containing JRM-28 and DTT confirms the existence of JRM-28a (trace), JRM-20 (trace), and JRM-28 (predominant, base peak at 561) in positive ion mode, (**C**) whereas the negative ion mode predominantly confirmed the generation of JRM-28a (**D**). The mass spectrum for JRM-20 with DDT displayed the base peak at 266 for JRM-28b (**E**). Luminescence assay for purified recombinant HDAC2 inhibition by JRM-28 (**F**) and JRM-20 (**G**) with and without DTT. HDAC2 assays were performed in triplicate, and the averages were plotted on the non-linear regression graph of the concentration-response equation using GraphPad Prism 8 software. In this assay, aminoluciferin and luciferase were coupled to deacetylase activity such that luminescence (arbitrary units, au) linearly reflected the rate of deacetylation. HDAC2 concentration (0.5 nM) and the dose of inhibitors were consistent in each reaction.

Accordingly, upon treatment with 350 mM DTT, JRM-28 was reduced to JRM-28a (Figure 2A) and JRM-28b, whereas the precursor molecule, JRM-20, generated only JRM-28b under the same condition, as confirmed by mass spectrometry (Figure 2B). The mass spectrum of the DTT-treated JRM-28 identified the predominant existence of JRM-28 (base peak 561 *m/z*) with traces of JRM-28b (266 *m/z*) and JRM-20 (529 *m/z*) in positive ion mode (Figure 2C). In the negative ion mode, we observed JRM-28a (296 *m/z*) as the major product (Figure 2D). On the other hand, DTT-treated JRM-20 displayed a base peak of 266 *m/z* in the mass spectrum, which represents the predominant existence of JRM-28b (Figure 2E).

While analyzing the effect of JRM-28 on HDAC2 inhibition, we observed that the addition of DTT markedly increased the inhibitory effect of JRM-28 by almost 200-fold (Figure 2F). The inhibition curve of JRM-28 in the presence of DTT resulted in an IC_50_ of 0.28 µM, whereas without DTT, the IC_50_ was 54.86 µM. Since, upon reduction, JRM-28 breaks down to JRM-28a and 28b, the HDAC2-inhibitory effect of each monomer is warranted. We monitored the dose-dependent inhibitory effect of JRM-28b by reducing JRM-20 (Figure 2G). The observed IC_50_ was 2.7 µM for JRM-28b, whereas JRM-20 in the non-reduced form displayed an IC_50_ value of 12.9 µM. Thus, it can be concluded that JRM-28a, but not 28b, is the most potent isoform that displays a strong HDAC2 inhibitory effect. We were not able to directly measure the HDAC activity of JRM-28a because the molecule spontaneously dimerized to form JRM-28.

**Figure 3:**
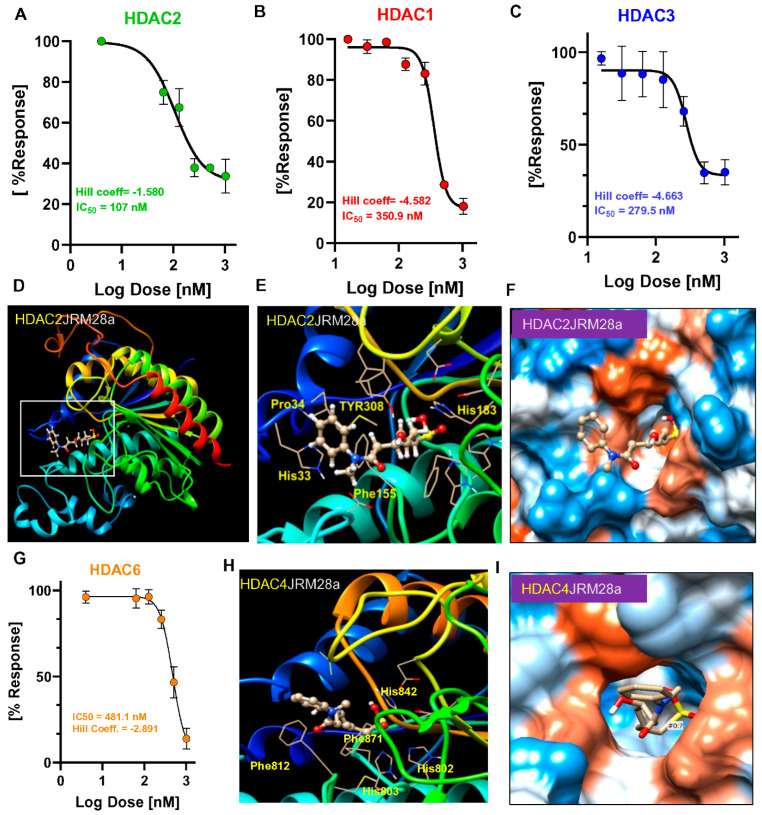
JRM-28 is an HDAC2 inhibitor. Fluorimetric inhibition analyses (Ex/Em = 480/528 nm) with purified (**A**) HDAC2, (**B**) HDAC1, and (**C**) HDAC3 were performed in the presence of increasing doses of JRM-28 (reduced with DTT). Respective IC_50_ and Hill slope were calculated from a non-linear inhibition curve derived from GraphPad Prism 9 software. Hill coefficients were found to be unstable in HDAC1 and HDAC3 binding curves with high IC_50_. The fluorescence value was normalized with DMSO control and then DTT control. (**D**) In silico binding assay displayed a low-resolution docking pose of JRM-28 with HDAC2. The PDB structure of rigid body docking analyses between HDAC2 and JRM-28a was visualized in Chimera 1.14 (UCSF) software. (**E**) The high-resolution image showed amino acid residues within an 11 Å distance of JRM-28a in a catalytic binding pocket. (**F**) The electrostatic potential surface displayed the charge distribution of HDAC2 protein around JRM-28a. Blue = positive charge, white = neutral, and red = negative charge. (**G**) HDAC6 inhibition assay was performed with increasing doses of JRM-28 (reduced) based on a fluorometric strategy (Ex/Em = 480/528 nm) followed by plotting the non-linear binding curve in GraphPad Prism 9 software. (**H**) In silico docking pose of JRM-28a with HDAC4 was visualized as a ribbon structure in UCSF Chimera 1.14 software. (**I**) Electrostatic potential surface display summarizing the charge distribution of HDAC4 around JRM-28a in its catalytic binding pocket.

The HDAC isoform selectivity of JRM-28 was investigated with class-I (HDAC1, 2, and 3) and class-II (HDAC6) HDACs using fluorescence-based enzyme assay. The fluorescence was normalized with DMSO control and adjusted in the presence of DTT. JRM-28 dose-dependently inhibited the activity of HDAC2 (Figure 3A) only, but not other class-I HDACs, including HDAC1 (Figure 3B) and HDAC3 (Figure 3C). In fact, reduced JRM-28 (JRM-28a+28b) displayed the characteristic sigmoidal binding curve (IC_50_ ~ 107 nM and Hill slope = −1.580) only with HDAC2, but not with HDAC1 (IC_50_ = 350.9 nM; Hill coefficient = −4.582) or 3 (IC_50_ = 279.5 nM; Hill coefficient = −4.663). Our in silico docking analysis of JRM-28a with HDAC2 (Figure 3D) predicted the complex formation of JR28a with HDAC2. The most stable pose was predicted based on the full-fitness score (−8.524 kcal/mol). According to that docking pose, the N-methyl acetanilide group of JRM-28a was found at the surface of the HDAC2 enzyme, the linker connected to the ZBG of JRM-28a at the catalytic side of HDAC2. The high-resolution wire representation of the docking image further identified surrounding amino acid residues, such as Gly 154, Phe 155, His 183, and Tyr 208 at 10 Å resolution (Figure 3E) in the catalytic site. Electrostatic potential surface representation displayed the hydrophobic and hydrophilic nature of the catalytic pocket (Figure 3F). Next, the interaction between JRM-28a and class-II HDAC HDAC6 was assessed, resulting in no appreciable inhibition (IC_50_ = 267.1 nM; Hill coefficient = −5.116) (Figure 3G). The in silico analysis of JRM-28a with another class-II HDAC, HDAC4, demonstrated (Figure 3H) that the ZBG of JRM-28a posed far from the catalytic pocket of HDAC4 [36], comprised of His802, His803, and His842, even though an electrostatic potential surface representation predicted the docking of JRM-28 in the catalytic pocket of HDAC4 (Figure 3I). Collectively, our results suggest that JRM-28a, the active component of JRM-28, serves as a strong inhibitor of HDAC2 while showing relatively weaker inhibition of other HDACs.

**Figure 4:**
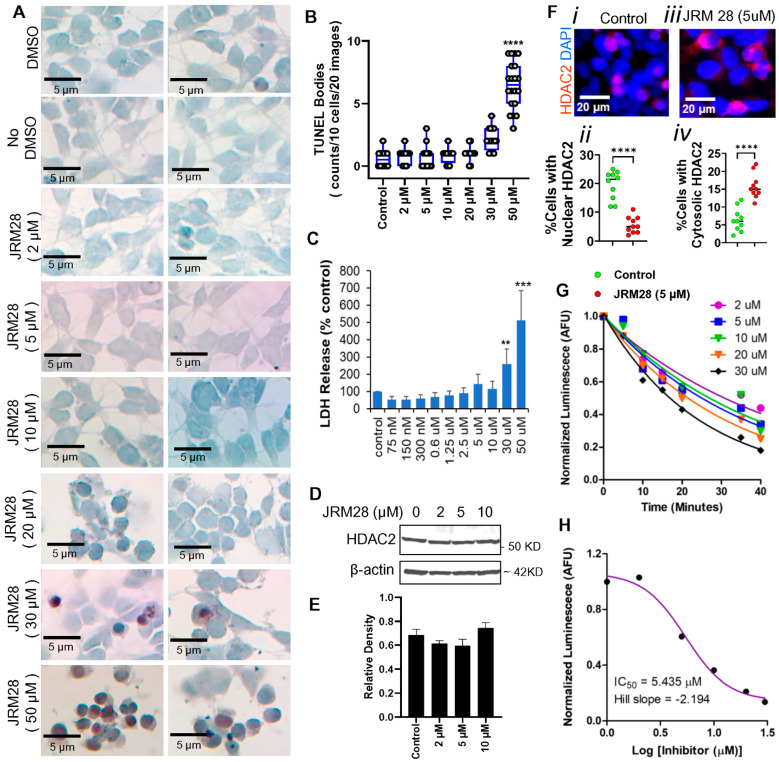
Exploring the cytotoxicity of JRM-28. SHSY5Y neurons were treated with different doses of JRM-28 for 24 h and then analyzed for different cytotoxicity assays. (**A**) Representative images are duplicate analyses of TUNEL staining in SHSY5Y neurons with increasing doses of JRM-28. TUNEL^+ve^ cells were detected as condensed brown bodies, whereas nuclei were stained with hematoxylin (*blue*). (**B**) TUNEL bodies were counted per 10 randomly selected cells in 20 different images. Results were plotted as a dotted boxplot, and the significance of the mean was analyzed by one-way ANOVA (single effector = treatment). **** *p* < 0.00001 versus DMSO control. (**C**) LDH (lactate dehydrogenase) release assay was performed in the supernatants after 24 h of treatment with increasing doses of JRM-28. One-way ANOVA resulted in ** *p* < 0.01 and *** *p* < 0.001 versus control. (**D**) Immunoblot (IB) analysis of HDAC2 in the total cell lysate of SHSY5Y cells treated with increasing doses of JRM-28 for 24 h. IB assay of β-actin was performed as a housekeeping protein (Supplementary Figure S18 for raw blot). (**E**) The band density of individual HDAC2 bands was assessed in ImageJ software (version 1.45) followed by normalization with respective β-actin band density. The relative densitometry was plotted by a bar diagram. (**F**) Immunofluorescence (IF) analysis of HDAC2 in SHSY5Y cells treated with DMSO (i) and 5 μM JRM-28 (iii). Nuclei were stained with DAPI. (ii) Cells with nuclear and (iv) cytosolic HDAC2 were counted in 10 independent images for both control and JRM-treated groups followed by scatter histogram analyses in percent scale. Unpaired t-test (**** *p* < 0.0001) was performed to measure the significance of means between groups. (**G**) Endogenous HDCA2 inhibition assay in the cell lysate of SHSY5Y cells treated with increasing doses of JRM-28. The assay was performed at a different time after the treatment with JRM-28. (**H**) Dose-dependent endogenous HDAC2 inhibition assay in SHSY5Y cells with increasing doses of JRM-28. A characteristic sigmoidal non-linear inhibitor binding curve was drawn in GraphPad Prism 9 software followed by measuring IC_50_ and Hill slope. Results are confirmed after three different experiments.

Next, JRM-28 in the presence of DTT was evaluated by cytotoxicity assays. A TUNEL assay was performed with SHSY5Y neuronal cells using increasing concentrations of JRM-28 in the presence of DTT. According to the assay (Figure 4A), higher doses of JRM-28 were found to be toxic, as 20 µM JRM-28 caused nuclear condensation (Figure 4A; *row 6 duplicates*), whereas 30 µM (Figure 4A; *row 7 duplicates*) and 50 µM (Figure 4A; *row 8 duplicates*) concentrations of JRM-28 induced significant cell death, as shown by increased TUNEL-positive inclusion bodies (brown spots) in the nuclei (blue) of SHSY5Y cells. Lower doses of JRM-28 up to 10 µM (Figure 4A; *top 5 rows in duplicate*) did not induce any cell death. The results were further confirmed by quantitative analysis (Figure 4B). Next, an LDH release assay (Figure 4C) demonstrated that 30 µM and 50 µM, but not lower concentrations of JRM-28, significantly induced LDH release by SHSY5Y cells. Next, we wanted to study the effect of lower concentrations of HDAC2 on the regulation of HDAC2 activity. An immunoblot (IB) analysis of HDAC2 (Figure 4D) followed by densitometric assessment (Figure 4E) revealed that lower concentrations, such as 2, 5, and 10 µM of JRM-28, did not upregulate the expression of HDAC2 in SHSY5Y cells compared to the control. Interestingly, subsequent immunocytochemical analyses (Figure 4F(i,iii)) together with quantitative estimation (Figure 4F(ii,iv)) indicated that 5 µM of JRM-28 significantly arrested the nuclear translocation of HDAC2, suggesting that low concentrations of JRM-28 inhibited the activation but not the expression of HDAC2. To further confirm this finding, we performed a luminescence-based HDAC2 activity assay in SHSY5Y cells with increasing doses of JRM-28. JRM-28 was not reduced by DTT during the treatment, as the cellular reductase and oxidoreductase enzymes are expected to reduce JRM-28 in its “active 28a/b” components. Shorter time points up to 45 min of treatment were selected because the HDAC2 activation occurs rapidly after treatment [37]. Accordingly, time-(Figure 4G) and dose-(Figure 4H) response analyses confirmed that 5 µM (IC_50_ = 5.435 µM) of JRM-28 significantly inhibited the HDAC2 activity in neuronal cells as early as 30 min of treatment. Collectively, these results identified that a 5 µM concentration of JRM-28 is optimal to achieve the maximum HDAC2 inhibition without causing a cytotoxic response.

### 3.2. JRM-28-Augmented Morphological Plasticity in Neurons

**Figure 5:**
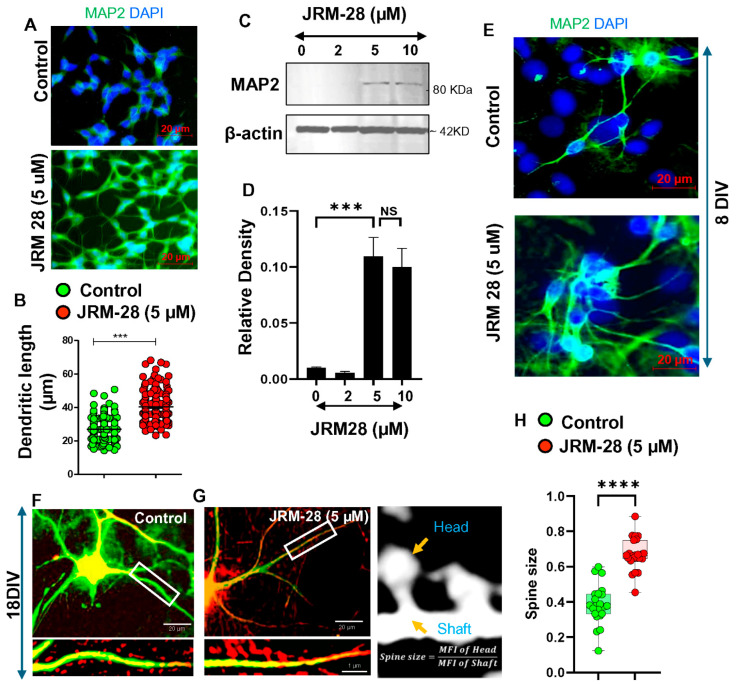
JRM-28 induces structural plasticity in neurons. SHSY5Y neurons and E18 mouse primary hippocampal neurons were grown in complete neurobasal media, treated with different doses of JRM-28 for 24 h, and then analyzed for dendritic morphogenesis by a different immunoassay. (**A**) After 24 h of seeding, JRM-28 was treated for an additional 24 h. After that, the immunofluorescence (IF) assay of dendritic marker MAP2 (green) was performed as described in the Methods section. Nuclei were stained with DAPI (blue). (**B**) The dendritic length was measured in 200 randomly selected neurons per group in ImageJ software. Briefly, scale bar calibration was performed after converting pixels to micrometer scale, followed by measuring dendritic length with the line tool. Two-tailed unpaired t-test was performed to test the significance of the means between groups, resulting in *** *p* < 0.0001 (*t* = 18.26; *df* = 398). (**C**) Immunoblot analysis of MAP2 (*top*) and beta-actin (*bottom*) in SHSY5Y cells treated with 2, 5, and 10 μM doses of JRM-28. (Supplementary Figure S18 for raw blot). (**D**) The band intensity was measured in ImageJ software and then normalized with the respective band density of beta-actin. Unpaired t-test resulted in *** *p* < 0.0001 vs. control, and ns = no significance. (**E**) E18 mouse primary neurons were grown in complete neurobasal media for 7 days in vitro (**DIV**). After that, 5 μM JRM-28 was treated for 24 h, followed by IF analysis of MAP2 (Green). Nuclei were stained with DAPI. For spine analysis, E18 neurons were grown in 18 DIV. Alexa 640-tagged Phalloidin staining (Red) was performed together with MAP2 (Green) to visualize dendritic spines on the dendrites in (**F**) control and (**G**) 5 μM JRM-28-treated neurons. The image was captured in 100× objective after oil immersion, followed by visualizing in LSM Zeiss image browsing software (version 4.2.0.121) at 400× magnification. (**H**) Spine size was measured following the measurement of the ratio between the MFI of the spine head and the MFI of the spine shaft, as described in the adjacent image and method. Spines were considered to be mature if the ratio was ≥ 0.6. Unpaired *t*-test generated **** *p* < 0.0001 versus control. Results are confirmed with mean ± SD of three different experiments.

Next, we studied lower doses of JRM-28 with respect to morphological plasticity in neurons. Dendritic morphogenesis, which directly correlates to the morphological plasticity of neurons [38], can be best evaluated by measuring the increase in length and branches of basal dendrites in neurons. Therefore, the effect of JRM-28 was monitored to explore the numbers of MAP2-ir dendrites in SHSY5Y human neuronal cultures. Briefly, SHSY5Y human neuronal cells were treated with 2, 5, and 10 μM concentrations of JRM-28 for 48 h, followed by immunofluorescence and immunoblot (IB) analyses of MAP2. Interestingly, 5 μM JRM-28 significantly stimulated dendritic pruning (Figure 5A), as indicated by increased numbers of MAP2-ir small appendages on neuronal cell bodies compared to the control. Moreover, a scatter histogram comparison (Figure 5B) of dendritic length between control and 5 μM JRM-28 (n = 200 per group) followed by an unpaired t-test analysis (t_1,398_ = 18.26; *** *p* < 0.001) confirmed that JRM-28 treatment indeed augmented the dendritic morphogenesis in neuronal cells. Moreover, an immunoblot analysis (Figure 5C) followed by densitometric analysis (Figure 5D) also indicated that 5 and 10 µM of JRM-28 upregulated the expressions of MAP2 in SHSY5Y cells. Next, the effect of JRM-28 on the upregulation of MAP2 was investigated in embryonic (E18) mouse primary hippocampal neurons (8 days in vitro). Accordingly, our immunocytochemical analysis (Figure 5E) demonstrated that 48 h of incubation with 5 µM JRM-28 significantly upregulated the synthesis of MAP2-ir basal dendrites in hippocampal neurons. Synthesis and maturation of dendritic spines on the surface of dendrites also regulated the morphological plasticity of hippocampal neurons. However, spine maturation can be best assessed in hippocampal neurons after 18 DIV growth condition. Therefore, E18 mouse hippocampal neurons were first dissected from E18 fetal brain, grown in complete neurobasal media for 18 days, and then treated with either DMSO control (Figure 5F) or 5 µM of JRM-28 (Figure 5G) for 48 h, followed by the evaluation of dendritic spines by MAP2 and phalloidin dual labeling. Dual immunostaining followed by a quantitative estimation of spine size by a scatter boxplot analysis (Figure 5H) indicated that JRM-28, but not the control, significantly induced the maturation of spines with increased head-to-shaft ratio (>0.6). Dendrites with more than 20 spines/10 µm length were considered for counting. On average, the head areas of mushroom spines were observed to be 0.009526 ± 0.0022 µm^2^ in control and 0.01384 ± 0.023 µm^2^ in JRM-28-treated condition (**** *p* < 0.0001 by unpaired t-test), further corroborating the role of JRM-28 on spine maturation. Collectively, our results suggest that JRM-28 augments structural plasticity via enhancing dendritic morphogenesis and spine maturation in hippocampal neurons.

### 3.3. JRM-28-Induced Morphological Plasticity via Transcriptional Activation of CREB

**Figure 6:**
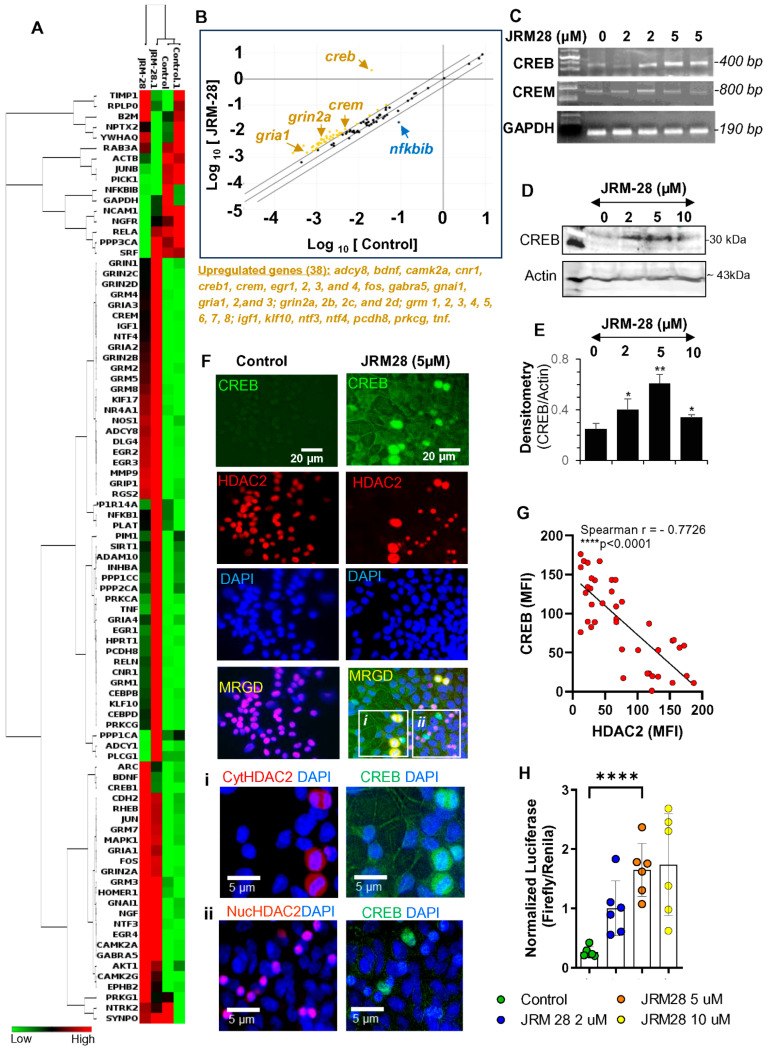
JRM-28 stimulates transcriptional activity of CREB via inhibition of HDAC2. SHSY5Y neurons were cultured, differentiated, and treated with different doses of JRM-28 for 24 h and then analyzed for PCR, immunoblot, dual IF, and firefly/renilla dual luciferase reporter assay. (**A**) A cDNA-based array for 88 CREB and CREB-dependent genes was performed in a commercially available array kit (RT^2^ Profiler PCR Array for human synaptic plasticity; Qiagen: Cat # PAHS-126-EZ). The Ct data were uploaded to Qiagen’s web analysis server. The result was summarized by (**A**) Heatmap and (**B**) scatter plot analyses. Thirty-eight genes, including CREB, were found to be upregulated. (**C**) Semi-quantitative RT-PCR analyses for CREB family of genes, including *creb* and *crem*. The 100 bp DNA ladder was run to identify the amplified products of these genes. The *gapdh* gene was included as the housekeeping gene (Supplementary Figure S18 for raw PCR gel). (**D**) Immunoblot analysis of CREB (*top*) and beta-actin (*bottom*) in SHSY5Y cells treated with increasing doses of JRM-28 (Supplementary Figure S18 for raw blot). (**E**) The band intensity was measured in ImageJ software and then normalized with the respective band density of beta-actin. * *p* < 0.05 (=0.0324), ** *p* < 0.01 (=0.0031), * *p* < 0.05 (=0.0477) vs. control. (**F**) Dual IF analysis of CREB (Green) and HDAC2 (Red) in control and 5 µM JRM-28-treated SHSY5Y cells. Nuclei were stained with DAPI. (*Inset*) Enclosed boxes magnified to estimate the relative distribution of CREB and HDAC2 for (*i*) cytosolic HDAC2 (CytHDAC2) and CREB as well as (*ii*) nuclear HDAC2 (NucHDAC2) and CREB. (**G**) A non-parametric Spearman correlation analysis in 40 randomly selected dual-stained cells with CREB and HDAC2 were considered for MFI calculations, followed by correlation statistics. The non-parametric correlation was considered once the dataset failed to pass the normality test. (**H**) The firefly *cre* luciferase assay was performed in 2, 5, and 10 µM JRM-28-treated SHSY5Y cells, normalized with renilla luciferase, and then offset with negative control. The normalized luciferase was plotted as a dotted histogram. One-way ANOVA demonstrated F3,20 = 9.731; **** *p* < 0.0005 (=0.0004). Results are confirmed with mean ± SD of three different experiments.

Previous reports suggest that the cAMP response element binding protein CREB plays a pivotal role in the induction of morphological plasticity [24,25]. Accordingly, RT^2^ Profiler PCR Array™ analysis of CREB family genes and CREB-dependent plasticity-associated genes (Figure 6A,B) indicated that 5 µM JRM-28 strongly upregulated the expressions of the creb gene and 37 other CREB-dependent genes. To further confirm the effect of JRM-28 on the expressions of CREB-family genes, we performed gene expression analyses (Figure 6A) of *creb* and its other family member *crem* (cAMP-responsive element modulator) in SHSY5Y neuronal cells. Accordingly, semi-quantitative RT-PCR analyses revealed that increasing concentrations of JRM-28 upregulated the mRNA expression of *creb* 5 µM, whereas 2 and 5 µM of JRM-28 failed to upregulate the expression of *crem*. Interestingly, the crem gene was found to be marginally but significantly (for significance, the cut-off > 2-fold compared to control) upregulated according to cDNA array analysis. However, according to scatter plot analysis (Figure 6B) followed by PCR study (Figure 6C), the expression of CREB was observed to be the most significant. Therefore, combining cDNA array and PCR data, we confirmed that the upregulation of CREB could be the direct target after HDAC2 inhibition by JRM-28. Nevertheless, immunoblotting (Figure 6D) followed by densitometric (Figure 6E) analysis further confirmed that increasing concentrations of JRM-28 upregulated the expression of CREB protein. These results suggest that suppression of HDAC2 activity could specifically upregulate the transcription of *creb* but not its other gene family members. To explore the effect of JRM-28-mediated inhibition of HDAC2 with respect to transcriptional upregulation of CREB, a series of experiments followed by quantitative analyses were carried out that indicated that the downregulation of HDAC2 by JRM-28 plays a critical role in the upregulation of CREB. First, a dual immunofluorescence analysis (Figure 6F) of HDAC2 and CREB demonstrated that the treatment with 5 µM JRM-28 significantly attenuated the expression of HDAC2 in the nuclei of SHSY5Y cells. Interestingly, analyzing the subcellular distribution of HDAC2 further indicated that the expression of CREB was only observed in cells with no HDAC2 or cells with inactive cytosolic HDAC2 (Figure 6F(i)). On the contrary, cells with active and nuclear HDAC2 displayed very marginal to no CREB immunoreactivity (Figure 6F(ii)), suggesting that the JRM-28-mediated inactivation of HDAC2 was associated with the upregulation of CREB expression. A non-parametric Spearman correlation analysis (Figure 6G) between the mean fluorescence intensity (MFI) of CREB and that of HDAC2 further demonstrated a strong negative correlation, suggesting that the suppression of HDAC2 expression by JRM-28 upregulated the expression of the CREB protein. Finally, a dual (renilla/firefly) CRE-luciferase reporter assay (Figure 6H) clearly indicated that increasing doses of JRM-28 upregulated the CREB-driven reporter activity, suggesting that the HDAC2 inhibition by JRM-28 not only upregulated the expression but also stimulated the transcriptional activity of CREB.

**Figure 7:**
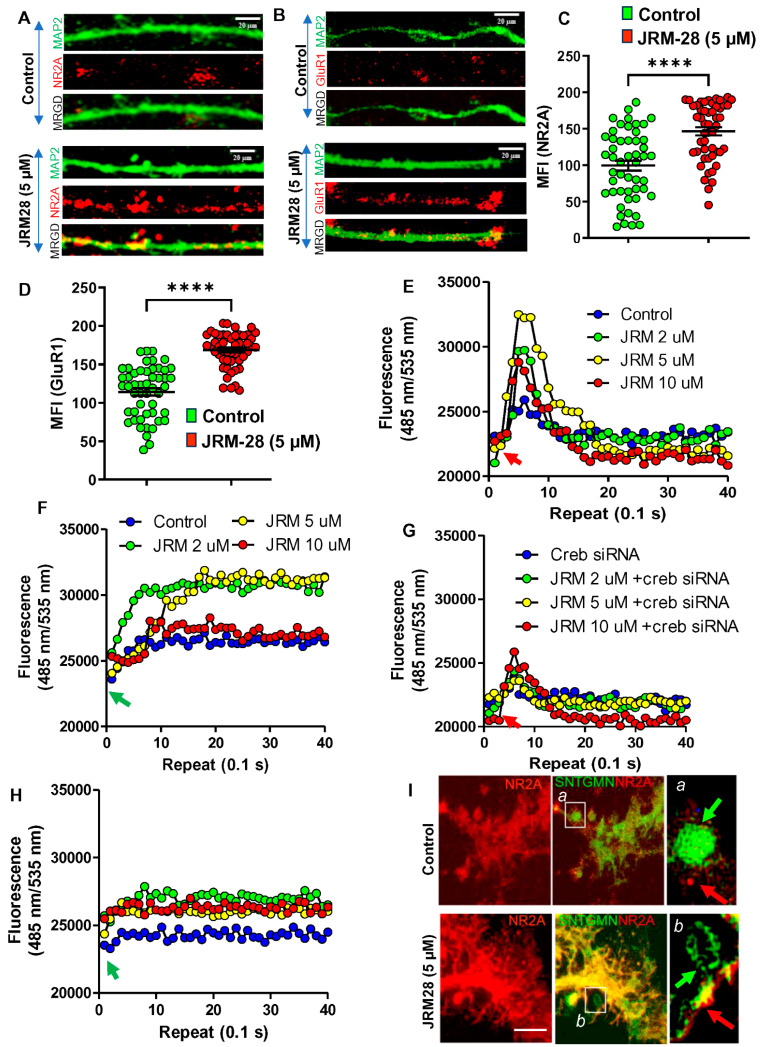
JRM-28 enhances post-synaptic calcium influx in E18 mouse primary hippocampal neurons. Mouse primary hippocampal neurons were generated from E18 fetal brains as described in the Methods section. After 18 days of differentiation, these neurons were treated with 5 µM of JRM-28 for 48 h and then analyzed for IF and calcium influx assay. The representative images demonstrate dual IF analyses of (**A**) MAP2 (green) plus NMDA receptor subunit NR2A (red); and (**B**) MAP2 (green) plus AMPA receptor subunit GluR1 (red). Nuclei were stained with DAPI (blue). (**C**) MFIs (mean fluorescence intensities) of NR2A and (**D**) GluR1 were calculated in ImageJ software in 50 different NR2A-ir and GluR1-ir receptors (as shown in red-colored dense bodies) per group and then plotted as dotted histograms. An unpaired *t*-test was adopted to test the significance of the means between groups, resulting in **** *p* < 0.0001 versus control (*t* = 5.334; *df* = 98) in NR2A analysis and **** *p* < 0.0001 versus control (*t* = 9.608; *df* = 98) for GluR1. (**E**) Ionotropic calcium assay was performed in hippocampal neurons with increasing doses of JRM-28. Recording started 0.1 s after the addition of 20 μM NMDA via a dual pump autoinjector, and the kinetic plot was recorded at Ex: Em 485:535 nm filter set at 0.1 s interval for 100 readings. The result was normalized with baseline and plotted as a linear scale. (**F**) AMPA-driven calcium influx assay in hippocampal neurons treated with 2, 5, and 10 μM of JRM-28. The recording was performed at a 0.1 s time interval for 100 repeats following the addition of 20 μM AMPA. (**G**) Hippocampal neurons were transfected with *creb* siRNA for 24 h, followed by treatment with different doses of JRM-28 for another 24 h. After that, an NMDA-sensitive calcium influx assay was performed. (**H**) AMPA-sensitive calcium entry assay was measured in *creb* siRNA-transfected hippocampal neurons after treatment with increasing doses of JRM-28. (**I**) Pre-synaptic protein synaptotagmin was dual labeled with NR2A. The enhanced interaction between these two proteins may represent augmented synaptic transmission. (a) In control cells, synaptotagmin-ir endocytic membrane fold (circular; green arrow) is found to be present near the post-synaptic NMDA receptor (red dot; NR2A; red arrow). (b) Synaptotagmin-ir endocytic membrane complex (green arrow) was found to be latched onto NR2A-ir (red arrow) NMDA receptor in JRM-28- (5 µM) treated neuron. Scale bar = 5 µm. Results are confirmed after three independent experiments.

The cDNA analyses demonstrated that JRM-28 treatment significantly upregulated genes that are essential for calcium ion transmission in neurons, such as genes for ionotropic NMDA receptors (grin2a, 2b, 2c, and 2d) and ionotropic AMPA receptors (gria 1, 2, and 3). NMDA- and AMPA-driven calcium influx through these ionotropic glutamate channels directly controls the morphological plasticity in post-synaptic hippocampal neurons [24]. Recent studies indicated that, upon activation [24,25], CREB upregulates the expressions of critical subunits of NMDA and AMPA receptors, such as Grin2a or NR2A, a critical subunit of the NMDA receptor, and Gria1 or GluR1, a functional subunit of the AMPA receptor. Accordingly, our IF studies followed by MFI analyses indicate that 5 μM JRM-28 upregulated expressions of both NR2A (Figure 7A,B) and GluR1 (Figure 7C,D) in E18 mouse hippocampal neurons. Since upregulations of proteins do not necessarily correlate with the function of the receptor, next, we were interested in studying if JRM-28 stimulated the NMDA- and AMPA-sensitive calcium influx in primary hippocampal neurons. A dose-dependent calcium influx study was performed in E18 hippocampal neurons at 100 repeats of 0.1 s intervals after 24 h of treatment with 2, 5, and 10 µM of JRM-28 treatment. Interestingly, a real-time calcium influx study indicated that 5 µM JRM-28 strongly upregulated the NMDA-(Figure 7E) and AMPA-(Figure 7F) sensitive calcium influx in mouse primary hippocampal neurons. The optimum dose of JRM-28 for the calcium influx was found to be 5 µM, as the higher concentration may desensitize the receptor to prevent uncontrolled calcium entry. According to previous reports [24,25], CREB plays a vital role in the augmentation of NMDA- and AMPA-driven ionotropic calcium influx through the dendritic spines of hippocampal neurons. Hence, the direct role of CREB was next evaluated in JRM-28-stimulated calcium influx through NMDA and AMPA receptors. Accordingly, the knocking down of *creb* gene expression by *creb* siRNA (Supplementary Figure S17) was found to significantly abrogate the NMDA- (Figure 7G) and AMPA- (Figure 7H) driven calcium influx in JRM-28-stimulated hippocampal neurons, suggesting the essential role of CREB in JRM-28-mediated ionotropic calcium influx in hippocampal neurons. The role of NMDA receptor-driven calcium influx in the augmentation of synaptic plasticity is often debated for its cytotoxic response. To nullify that possibility, we performed a dual immunolabeling analysis of pre-synaptic protein synaptotagmin and post-synaptic NMDA receptor protein NR2A (Figure 7I) in 18 DIV hippocampal neurons. During calcium transmission through the vesicle, the synaptotagmin-ir pre-synaptic membrane fold is expected to interact with the NR2A-ir post-synaptic NMDA receptor. Interestingly, we observed that JRM-28 treatment, but not control, facilitates the interaction of the endocytic membrane with the post-synaptic NMDA receptor, suggesting that 5 µM JRM-28 treatment augments dendritic strength via induction of NMDA-dependent calcium current.

### 3.4. JRM-28 Augments Morphological Plasticity in iPSC-Derived AD Neurons via Activation of CREB

**Figure 8:**
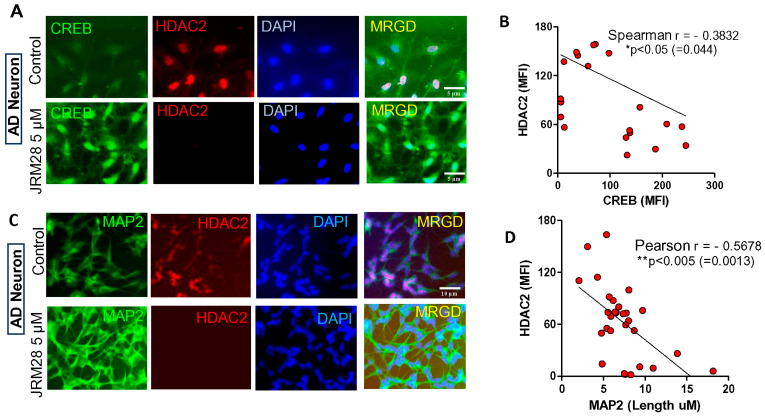
The effect of JRM-28-mediated suppression of HDAC2 on the upregulation of CREB and MAP2 in AD neurons with A246E *psen1* mutation. AD neurons were differentiated from iPSC-derived neural stem cells (NSCs) in a conditional neurobasal media as described in the Methods section. AD neurons were stimulated with 5 µM of JRM-28 for 48 h and then immunolabeled. (**A**) The representative image exhibits a dual IF analysis of CREB (green) and HDAC2 (red). Nuclei were stained with DAPI (blue). (**B**) A non-parametric Spearman correlation analysis of mean fluorescence intensities (MFIs) was performed between CREB and HDAC2. The resultant scatter plot displays a moderate negative correlation (* *p* < 0.05). A total of 30 random neurons were selected from 6 different images (3 images per group) in this analysis. MFI was calculated in ImageJ software, the raw values were recorded, and the derived dataset was analyzed for the normality test in GraphPad Prism 8 software. Spearman correlation analysis was decided once the dataset failed to pass the D’Agostino–Pearson normality test. (**C**) A representative dual IF image of MAP2 (green) and HDAC2 (red). Nuclei were labeled with DAPI (blue). (**D**) A parametric Pearson correlation statistic of MFIs between HDAC2 and MAP2 was performed after the normality test was successfully executed in GraphPad Prism 8 software. A total of 30 neurons from six independent images (three images/group) were selected in this analysis. Results are confirmed after three different experiments.

**Figure 9:**
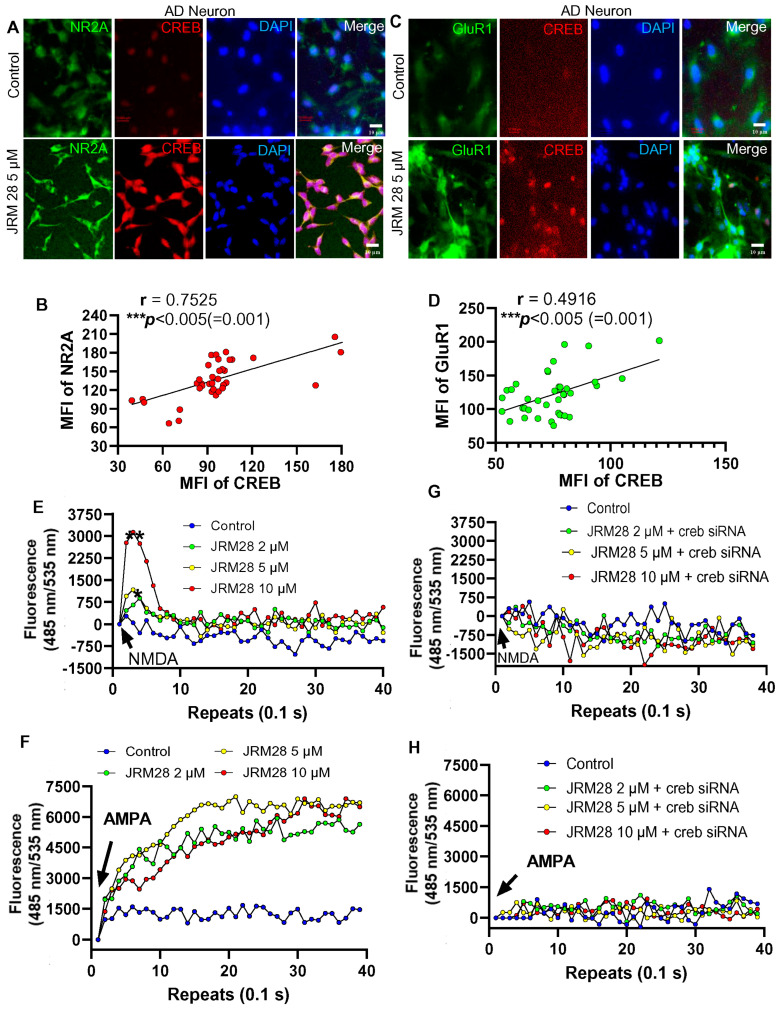
The effect of JRM-28-mediated activation of CREB on the upregulation of NR2A, GluR1, and induction of ionotropic calcium entry in AD neurons. AD neurons were differentiated from iPSC-derived neural stem cells (NSCs) as described under the Methods section, followed by treatment with 5 µM of JRM-28 for 48 h, and then analyzed for IF and calcium influx assay. (**A**) The representative image exhibits a dual IF analysis of NR2A (green) and CREB (red). Nuclei were stained with DAPI (blue). (**B**) A non-parametric Spearman correlation analysis of mean fluorescence intensities (MFIs) was performed between CREB and NR2A. The resultant scatter plot displays a strong positive correlation (*** *p* < 0.005). A total of 30 random neurons were selected from six different images (three images per group) in this analysis. MFI was calculated in ImageJ software, the raw values were recorded, and the derived dataset was analyzed for the normality test in GraphPad Prism 8 software. Spearman correlation analysis was decided once the dataset failed to pass the D’Agostino–Pearson normality test. MFI measurement was restricted to cell bodies only. (**C**) A representative dual IF image of GluR1 (green) and CREB (red). Nuclei were labeled with DAPI (blue). (**D**) A parametric Pearson correlation statistic of MFIs between CREB and GluR1 was performed after the normality test was successfully executed in GraphPad Prism 8 software. A total of 30 neurons from six independent images (three images/group) were selected in this analysis. Results are confirmed after three different experiments. (**E**) Ionotropic calcium assay was performed in AD neurons with increasing doses of JRM-28. Recording started 0.1 s after the addition of 20 μM NMDA via a dual pump autoinjector, and the kinetic plot was recorded at Ex: Em 485:535 nm filter set at 0.1 s interval for 100 readings. The result was normalized with baseline and plotted. * *p* < 0.05 and ** *p* < 0.01 versus maximum fluorescence value of control reading. (**F**) AMPA-driven calcium influx assay in AD neurons treated with 2, 5, and 10 μM of JRM-28. The recording was performed at a 0.1 sec time interval for 100 repeats following the addition of 20 μM AMPA. (**G**) AD neurons were transfected with the *creb* siRNA for 24 h, followed by treatment with different doses of JRM-28 for another 24 h. After that, an NMDA-sensitive calcium influx assay was performed. (**H**) AMPA-sensitive calcium entry assay was measured in *creb* siRNA-transfected AD neurons after the treatment with increasing doses of JRM-28. Results are confirmed after three independent experiments.

Alzheimer’s disease (AD) is a progressive neurodegenerative disease in the midbrain that severely impairs the hippocampal function of learning and memory [39]. During the progression of AD, neuronal cells of the cortex and hippocampus suffer loss in synaptic function and dendritic morphologies [40,41], resulting in neurocognitive impairments. Next, we examined the effect of JRM-28 on improving the morphological plasticity in AD neurons. These neurons were derived from neural stem cells (NSCs) of a 31-year-old female AD patient having the A246E pathogenic mutation at the *psen1* gene. These NSCs were derived from induced pluripotent stem cells (iPSCs) after reprogramming of skin fibroblast cells and obtained commercially from AXOL Bioscience (Edinburgh, UK). NSCs were maintained and differentiated into neurons as described in the Methods section. Next, we were interested in studying if JRM-28-mediated suppression of HDAC2 upregulated the expression of CREB in these AD neurons. Interestingly, a dual IF assay of CREB and HDAC2 (Figure 8A) supported by a negative Pearson correlation analysis (Figure 8B) indicated that upon treatment with 5 μM JRM-28 for 24 h, iPSC-derived AD neurons exhibited a strong upregulation of CREB coupled with a significant downregulation of HDAC2. Interestingly, a similar dual IF analysis (Figure 8C) of HDAC2 and MAP2 indicated that JRM-28-mediated downregulation of HDAC2 was found to increase the length of MAP2-ir basal dendrites in these AD neurons. Moreover, a Pearson correlation analysis (Figure 8D) between the MFI of HDAC2 and the dendritic length of basal dendrites reiterated that the downregulation of HDAC2 is indeed involved in the augmentation of dendritic morphogenesis in these AD neurons. To further evaluate the effect of CREB on JRM-28-induced morphological plasticity, a dual IF analysis of CREB was performed with NMDA receptor subunit NR2A (Figure 9A) or AMPA receptor subunit GluR1 (Figure 9C). Interestingly, these immunolabeling studies followed by correlation analyses (Figure 9B, D) clearly indicated that JRM-28-mediated upregulation of CREB truly augmented the expressions of ionotropic glutamate receptors in dendrites of AD neurons. While studying the dose-dependent effect of JRM-28 on ionotropic calcium influx, we observed significant upregulations of both NMDA- (Figure 9E) and AMPA- (Figure 9F) driven calcium entry in AD neurons. According to that analysis, JRM-28 displayed a dose-dependent increase of NMDA-driven calcium entry, which was significantly high at a 5 µM dose with a maximum at 10 µM concentration. On the other hand, we observed a strong AMPA-driven calcium influx at 5 µM dose. To confirm the direct role of CREB in JRM-28-mediated calcium entry, we performed a similar calcium entry assay in creb siRNA-transfected AD neurons. Interestingly, knocking down of CREB expression by *creb* siRNA strongly attenuated both NMDA- (Figure 9G) and AMPA- (Figure 9H) induced calcium entry in JRM-28-treated AD neurons, suggesting the essential role of CREB in JRM-28-mediated calcium entry in AD neurons.

Taken together, our current study highlights the discovery of a novel HDAC2 inhibitor, JRM-28, and its potential role in inducing neuronal plasticity via activation of CREB.

## 4. Discussion

### 4.1. JRM-28 Is a Selective HDAC2 Inhibitor

Previous studies indicated that suppression of HDAC2 activity by small-molecule therapeutics might display beneficial effects in the improvement of neurocognitive function [42,43] and the amelioration of AD pathologies [44], such as the removal of amyloid plaque burden [45] and the attenuation of Tau pathologies [46]. Although the molecular mechanism is still unclear, the role of HDAC2 suppression in inducing morphological plasticity, such as promoting synaptic transmission and augmenting dendritic morphogenesis, should be studied with prime importance. However, the major challenge is the highly overlapping structural similarities of all class-I HDACs, which makes it difficult to identify a selective inhibitor of HDAC2. In our current manuscript, we report the synthesis and characterization of a novel HDAC2 inhibitor termed JRM-28, followed by evaluating its effect in promoting morphological plasticity in hippocampal neurons. JRM-28 is a macrocyclic compound, which is designed and synthesized to satisfy the structural determinants of a classical HDAC inhibitor with an N-methyl acetalide “cap” group, a lipophilic “linker”, and a sulfur-containing ZBG. Originally, JRM-28 was synthesized as a prodrug, which is a dimer of two monomeric units with a disulfide linkage, and one sulfur unit was oxidized twice. Based on ^13^CNMR, ^1^HNMR, and LC-MS analyses, it was found that upon reduction, JRM-28 was derivatized to JRM-28a, a monomer with a sulfonate (-SO_2_H) group, and JRM-28b, another monomer with a sulfhydryl (-SH) group. Both JRM-28a and 28b were evaluated for HDAC2 inhibition by luminescence and fluorimetric inhibitor screening strategies. A dose-responsive ligand binding assay with purified HDAC2 revealed that JRM-28a was a stronger inhibitor (IC_50_ = 0.28 µM) of HDAC2 than JRM-28b (IC_50_ = 2.713 µM). Moreover, a fluorimetric binding assay with purified HDAC1, HDAC2, HDAC3, and HDAC6, followed by the comparative analyses of IC_50_ values and Hill slopes of sigmoidal binding curves, further indicated that JRM-28a could be a selective inhibitor only for HDAC2, but not other HDACs. An in silico docking simulation study once more indicated that JRM-28a was strongly docked in the catalytic pocket of HDAC2, whereas weaker docking was observed while analyzing the interaction between JRM-28a and HDAC4, a class-II HDAC.

### 4.2. JRM-28 Displays a Strong Neuroprotective Response by Inducing Morphological Plasticity in Neurons

Before exploring the neuroprotective properties of JRM-28, different cellular assays confirmed that a 5 μM dose of JRM-28 significantly attenuated the activation of HDAC2 without causing any cell death. While exploring the molecular changes in neurons, the most striking observation was the upregulated synthesis of apical dendrites in SHSY5Y neurons upon treatment with 5 μM of JRM-28. Dendritic morphogenesis is an integral mechanism of morphological plasticity. Being intrigued by the initial observation, we repeated the same experiment in E18 mouse primary hippocampal neurons, which further demonstrated that JRM-28 indeed induced dendritic morphogenesis in hippocampal neurons. When applied to the fully differentiated hippocampal neurons, we observed that JRM-28 strongly induced the maturation of dendritic spines. Dendritic spines are the home of ionotropic glutamate receptors, which play a direct role in post-synaptic neurotransmission. Accordingly, we observed that JRM-28 not only upregulated expressions but also dose-dependently induced calcium influx through these receptors.

### 4.3. The JRM-28-Mediated Inactivation of HDAC2 Triggers the Transcriptional Activation of CREB

A growing body of evidence [24,25] suggests that the transcriptional activation of CREB plays a central role in regulating morphological plasticity [6]. Several plasticity-associated genes contain multiple cAMP response elements (CRE) in their promoter region [24,47,48,49]. While studying the role of JRM-28 in the regulation of CREB, we observed that JRM-28 dose-dependently induced the expression and the transcriptional activity of CREB. Moreover, we observed a strong association between the JRM-28-mediated inactivation of HDAC2 and the upregulation of CREB, suggesting the direct role of HDAC2 inactivation in the augmentation of CREB’s transcriptional activity. However, two other members of CREB family proteins, including activating transcription factor-1 (ATF-1) and cAMP responsive element modulator (CREM), are also activated in response to several CREB-inducing molecular factors, including cAMP, calcium, and mitogenic stimuli [50]. Therefore, along with the evaluation of the transcriptional activity of CREB, the effects of JRM-28 on the regulations of CREM and ATF-I were also scrutinized. Interestingly, JRM-28-mediated inactivation of HDAC2 was observed to upregulate the expression of CREB only, not CREM or ATF-1.

### 4.4. JRM-28 Upregulates Neuronal Plasticity in AD Neurons

It is believed that neuronal plasticity decreases in neurons of the AD brain. Recent studies indicate that the markers of morphological plasticity, such as spine density [51], dendritic arbors [52], and post-synaptic neurotransmission [53], are severely impaired in the hippocampal neurons of AD patients. Therefore, identifying a therapeutic strategy to induce morphological plasticity in AD is an important area of research. To address this challenge, JRM-28 was applied to the AD neurons generated from iPSC-derived NSCs of a 31-year-old female AD patient with an A246E mutation at the *psen1* gene.

Here, we delineated that upon 48 h of stimulation with JRM-28, the molecular markers of post-synaptic neurons such as NR2A and GluR1 were strongly upregulated in AD neurons. As a result, we observed that JRM-28 stimulated the ionotropic calcium entry in cultured AD neurons, suggesting that, similar to primary hippocampal neurons, JRM-28 is also capable of inducing neuronal plasticity in AD neurons. While exploring the molecular mechanism, we observed that JRM-28-mediated inactivation of HDAC2 induced the expression of CREB in AD neurons. The elevated nuclear translocation of CREB demonstrated its elevated transcriptional activity, and, as a result, the expressions of CREB-dependent proteins such as NR2A and GluR1 were upregulated. Moreover, JRM-28 was found to stimulate the NMDA- and AMPA-driven calcium entry in AD neurons, suggesting that JRM-28 not only upregulated the expressions of proteins but also induced post-synaptic neurotransmission. The direct role of CREB in JRM-28-mediated neurotransmission was validated by a gene silencing study when a siRNA-mediated ablation of CREB strongly inhibited JRM-28-stimulated NMDA- and AMPA-driven calcium influx in AD neurons.

In summary, the current literature highlights the discovery of a novel small molecular compound that selectively inhibits HDAC2 but not other HDACs. By inhibiting HDAC2, JRM-28 stimulated the transcriptional activity of CREB and subsequently augmented the downstream events of structural plasticity, including dendritic morphogenesis, spine maturation, and neurotransmission through ionotropic glutamate receptors.

## Figures and Tables

**Table 1:** HDAC proteins, antibodies, and application.

Recombinant Proteins/Antibodies	Catalog #	Vendor	Application
**HDAC1 protein**	50051	BPS Bioscience, San Diego, CA, USA.	Luminescence/fluorescence binding assay
**HDAC2 protein**	50002	BPS Bioscience,	Luminescence/fluorescence binding assay
**HDAC3 protein**	50003	BPS Bioscience	Luminescence/fluorescence binding assay
**HDAC6 protein**	50056	BPS Bioscience.	Luminescence/fluorescence binding assay
**Anti-HDAC2 ab**	MA5-32101	Invitrogen, Waltham, MA, USA	IB (1:500), IF (1:250)
**Anti-MAP2 ab**	13-1500	Invitrogen	IB (1:500), IF (1:250)
**Anti-CREB ab**	MA1-083	Invitrogen	IB (1:500), IF (1:250)
**Anti-NR2A ab**	28525-1-AP	Proteintech Group, Rosemont, IL, USA	IB (1:500), IF (1:250)
**Anti-GluR1 ab**	MA5-32344	Invitrogen	IB (1:500), IF (1:250)

**Table 2:** List of creb family primers used in RT-PCR.

Gene	Forward Primer	Reverse Primer	Product Length
*creb*	ATTGCCACATTAGCCCAGGT	GTGTAGGAAGTGCTGAAGTCTC	400
*atf-1*	GCCCCCAAATAAAAAGACGAGG	TGCTGGGCACAAGTATCTGC	355
*crem*	GTGAGCTGAGATCAGGCACCAG	TTCAAGCACAGCCACACGAT	806

**Table 3:** List of antibodies used for western blot (WB) and immunofluorescence (IF).

Antibody	Vendor	Catalog#	Host	Application	Dilution
NR2A/GRIN2A Polyclonal antibody	Proteintech	28525-1-AP	Rabbit	WB, IF	1:1000 (WB) 1:250 (IF)
GluR1 Monoclonal Antibody	Invitrogen	MA5-32344	Rabbit	WB, IF	1:500 (WB) 1:250 (IF)
MAP2 Monoclonal Antibody	Invitrogen	13-1500	Mouse	WB, IF	1:500 (WB) 1:250 (IF)
CREB Monoclonal Antibody	Invitrogen	MA1-083	Mouse	WB, IF	1:500 (WB) 1:250 (IF)
Synptotagmin mo	Invitrogen	MA1-25568	Mouse	IF	1:250 (IF)
beta Actin Monoclonal Antibody	Invitrogen	MA5-15739	Mouse	WB	1:1000 (WB)
IRDye^®^ 680 anti-Mouse 2° Antibody	Li-Cor	926-68072	Donkey	WB	1:500 (WB)
IRDye^®^ 680 anti-Rabbit 2° Antibody	Li-Cor	926-68073	Donkey	WB	1:500 (WB)
IRDye^®^ 800 anti-Rabbit 2° Antibody	Li-Cor	926-32213	Donkey	WB	1:500 (WB)
IRDye^®^ 800 anti-Mouse 2° Antibody	Li-Cor	926-32212	Donkey	WB	1:500 (WB)
Goat anti-Rabbit 2° Antibody, TRITC	Invitrogen	T-2769	Goat	IF	1:200
Goat anti-Mouse, Alexa Fluor™ 488 2° Antibody	Invitrogen	A-21121	Goat	IF	1:200

## Data Availability

The electronic raw data are uploaded in Mendeley and can be available shortly by this link: https://data.mendeley.com/v1/datasets/yjgk38gp32/1.

## Supplementary Materials

The following supporting information can be downloaded at: https://www.mdpi.com/article/10.3390/cells13231964/s1, Figure S1: ^1^H-NMR of 3-(Tritylthio)propanal (Compound #3 mentioned in Figure 1A); Figure S2: ^13^C-NMR of 3-(Tritylthio)propanal (Compound #3 mentioned in Figure 1A); Figure S3: ^1^H-NMR of (*E*)-5-(tritylthio)pent-2-enal (Compound #5 mentioned in Figure 1A); Figure S4: ^13^C-NMR of (*E*)-5-(tritylthio)pent-2-enal (Compound #5 mentioned in Figure 1A); Figure S5: ^1^H-NMR of (*E*)-3-hydroxy-*N*-methyl-*N*-phenyl-7-(tritylthio)hept-4-enamide (Compound #7 mentioned in Figure 1A); Figure S6: ^13^C-NMR of (*E*)-3-hydroxy-*N*-methyl-*N*-phenyl-7-(tritylthio)hept-4-enamide (Compound #7 mentioned in Figure 1A); Figure S7: ^1^H-NMR of (4*E*,4’*E*)-7,7’-disulfanediylbis(3-hydroxy-*N*-methyl-*N*-phenylhept-4-enamide) (Compound #8 mentioned in Figure 1A); Figure S8: ^13^C-NMR of (4*E*,4’*E*)-7,7’-disulfanediylbis(3-hydroxy-*N*-methyl-*N*-phenylhept-4-enamide) (Compound #8 mentioned in Figure 1A); Figure S9: HRMS of (4*E*,4’*E*)-7,7’-disulfanediylbis(3-hydroxy-*N*-methyl-*N*-phenylhept-4-enamide) (Compound #8 mentioned in Figure 1A); Figure S10: ^1^H-NMR of *S*-((*E*)-5-hydroxy-7-(methyl(phenyl)amino)-7-oxohept-3-en-1-yl) (*E*)-5-hydroxy-7-(methyl(phenyl)amino)-7-oxohept-3-ene-1-sulfinothioate (Compound #9 mentioned in Figure 1A); Figure S11: ^13^C-NMR of *S*-((*E*)-5-hydroxy-7-(methyl(phenyl)amino)-7-oxohept-3-en-1-yl) (*E*)-5-hydroxy-7-(methyl(phenyl)amino)-7-oxohept-3-ene-1-sulfinothioate (Compound #9 mentioned in Figure 1A); Figure S12: HRMS of *S*-((*E*)-5-hydroxy-7-(methyl(phenyl)amino)-7-oxohept-3-en-1-yl) (*E*)-5-hydroxy-7-(methyl(phenyl)amino)-7-oxohept-3-ene-1-sulfinothioate (Compound #9 mentioned in Figure 1A); Figure S13: ^1^H-NMR of *S*-((*E*)-5-hydroxy-7-(methyl(phenyl)amino)-7-oxohept-3-en-1-yl) (*E*)-5-hydroxy-7-(methyl(phenyl)amino)-7-oxohept-3-ene-1-sulfonothioate (JRM-28 mentioned in Figure 1A); Figure S14: ^13^C-NMR of *S*-((*E*)-5-hydroxy-7-(methyl(phenyl)amino)-7-oxohept-3-en-1-yl) (*E*)-5-hydroxy-7-(methyl(phenyl)amino)-7-oxohept-3-ene-1-sulfonothioate (JRM-28 mentioned in Figure 1A); Figure S15: HRMS of *S*-((*E*)-5-hydroxy-7-(methyl(phenyl)amino)-7-oxohept-3-en-1-yl) (*E*)-5-hydroxy-7-(methyl(phenyl)amino)-7-oxohept-3-ene-1-sulfonothioate (JRM-28 mentioned in Figure 1A); Figure S16: Predicted schema of DTT-mediated reduction of JRM-28; Figure S17: Efficacy of creb siRNA. (A) SHSY5Y cells were treated with 0.1 µg of creb siRNA as described under method section. After 24 h, cells were treated with JRM28 and after another 24 h cells were fixed and stained with CREB antibody. Nuclei were stained with DAPI; Figure S18: Raw western blot and RT-PCR images used in the manuscript. References [54,55,56,57] are cited in the Supplementary Materials.

## Abbreviations

HDAC2 = Histone deacetylase 2; CREB = cAMP responsive element binding protein 2; NR2A = NMDA receptor subunit 2A; GluR1 = AMPA-sensitive glutamate receptor 1; IF = Immunofluorescence; IB = Immunoblot; HDACi = HDAC inhibitor; Et_3_N = triethyl amine; CH_2_Cl_2_ = dichloromethane; C_6_H_6_ = benzene; LDA = lithium diisopropylamide; THF = tetrahydrofuran; I_2_ = iodine; NaOAc = sodium acetate; MeOH = methanol; mCPBA = meta-chloroperoxybenzoic acid.
