# Supplementary material for: JRM-28, a Novel HDAC2 Inhibitor, Upregulates Plasticity-Associated Proteins in Hippocampal Neurons and Enhances Morphological Plasticity via Activation of CREB: Implications for Alzheimer’s Disease
Source: Cells. 2024 Nov 27;13(23):1964. doi: 10.3390/cells13231964 (PMC11640089; doi:10.3390/cells13231964)
Supplement: Supplementary file 1 [file cells-13-01964-s001.zip › cells-3226162-supplementary.pdf]

Supplementary Information:

JRM-28, a Novel HDAC2 Inhibitor, Upregulates Plasticity-Associated Proteins in Hippocampal Neurons and Enhances Morphological Plasticity via Activation of CREB: Implications for Alzheimer's Disease

A. F. M. Towheedur Rahman [1], Sarojini Bulbule [1,2], Jawad Bin Belayet [1], Anna Benko [1,3], Carl Gunnar Gottschalk [1,2], David N. Frick [1,3], Leggy A. Arnold [1,3], M. Mahmun Hossain [1,3,*] and Avik Roy [1,2,3,4,*]

1 Department of Chemistry and Biochemistry, University of Wisconsin-Milwaukee, 2000 E Kenwood Blvd, Mil-waukee, WI 53211, USA.; rahman25@uwm.edu (A.F.M.T.R.); sb@simmaron.com (S.B.); jbealyet16@gmail.com (J.B.B.); abenko@uwm.edu (A.B.); ggottschalk@simmaron.com (C.G.G.); frickd@uwm.edu (D.N.F.); ar-nold2@uwm.edu (L.A.A.)
2 Simmaron Research Institute, 948 Incline Way, Incline Village, NV 89451, USA
3 Milwaukee Institute for Drug Discovery, 2000 E Kenwood Blvd, Milwaukee, WI 53211, USA
4 Simmaron Research and Development Laboratory, University of Wisconsin-Milwaukee, Chemistry Building, 2000 E Kenwood Blvd, Suite # 320, Milwaukee, WI 53211, USA
* Correspondence: mahmun@uwm.edu (M.M.H.); avikroy@uwm.edu (A.R.); Tel.: +1-(414)-795-0383 (M.M.H.); +1-(773)-263-8182 (A.R.)

Supplementary Method:

General Methods and Experimental

2.6.1. General Consideration

Under dry nitrogen conditions, unless otherwise stated, all reactions were performed following standard Schlenk techniques. Prior use, The reaction vessels were filled with nitrogen and dried under vacuum using a flame. Reagents and solvents were purchased from Sigma-Aldrich, Milwaukee. All ^{1}H and ^{13}C NMR spectra were recorded in $CDCl_3$ (internal standard: 7.26 ppm, ^{1}H; 77.16 ppm, ^{13}C) at room temperature with a Burker 500 MHz spectrometer. The chemical shifts are given in parts per million (ppm), while the coupling constants are given in Hertz (Hz). A singlet, a doublet, a triplet, a quadruplet, and a multiplet was abbreviated as s, d, t, q, and m respectively. ^{1}H NMR. ^{1}H NMR, ^{13}C NMR, and high-resolution mass spectrometry (HRMS) techniques were employed to characterize all new compounds. HRMS were obtained using electrospray ionization (ESI) technique. For column chromatography, silica gel (35-70 microns) was used. Thin layer chromatography (TLC) was performed on aluminium-backed plates precoated (0.25 mm) with Silica Gel 60 F254 with a suitable solvent system and was visualized using UV fluorescence and/or iodine chamber.

2.6.2. Experimental:

Synthesis of 3-(Tritylthio)propanal, 3:

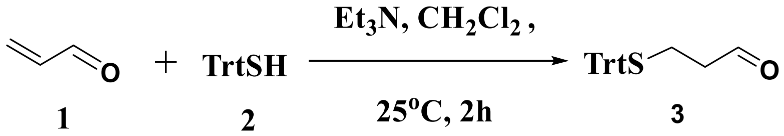

A round bottom flask was charged with triphenylmethyl mercaptan, compound #**2** (5.0 g, 18.1 mmol). The flask was put under argon and the contents of the flask were dissolved in dichloromethane (50 mL). Triethylamine (3.0 mL, 21.7 mmol, 1.2 equiv.) was added to the mixture and was stirred for additional 10 minutes. Acrolein, compound #**1** (1.2 mL, 18.1 mmol) was added to the mixture dropwise and was stirred for 2 hours. Completion of the reaction was confirmed by thin-layer chromatography (TLC) using 15% ethyl acetate/hexane. The crude reaction was passed through a silica plug and then concentrated in-vacuo. Product# **3** was purified by recrystallization with 10% ethyl acetate/hexane and collected 5.3 g with 88% yield 3. Product# **3** was confirmed by comparing spectra to known NMR. ^{1}H NMR (500 MHz, $CDCl_3$) δ 9.58 (t, *J* = 1.6 Hz, 1H), 7.45 (dt, *J* = 5.1, 2.0 Hz, 6H), 7.31 (ddd, *J* = 14.4, 7.4, 5.1 Hz, 6H), 7.25 (ddd, *J* = 7.2, 4.0, 1.4 Hz, 3H), 2.51 – 2.46 (m, 2H), 2.42 – 2.37 (m, 2H).; ^{13}C NMR (126 MHz, $CDCl_3$) δ 200.33, 144.53, 129.56, 127.99, 126.78, 67.01, 42.69, 24.42.

Synthesis of (*E*)-5-(tritylthio)pent-2-enal, 5:

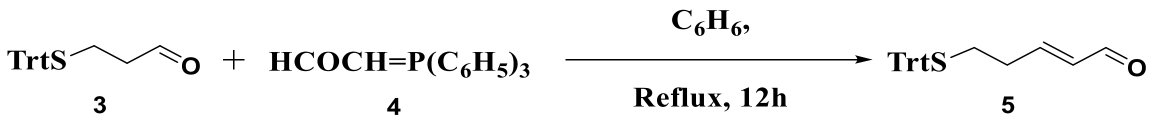

A round bottom flask was charged with product#**3** (5.0 g, 15.0 mmol, 1 equiv.) and 2-(triphenylphosphoranylidene)acetaldehyde, product#**4** (5.3 g, 16.6 mmol, 1.1 equiv.). The flask was put under argon and the contents of the flask were dissolved in benzene (100 mL). The solution was then refluxed overnight. When all starting materials were found to have disappeared, the reaction mixture was allowed to cool to room temperature and was then concentrated in vacuo. The crude product was separated via column chromatography and the column was run with an ethyl acetate/ hexane (1:7) solution until the product spot eluted. Then the product was purified by recrystallization with toluene to give 3.8 g (70%) of pure product **5**. Compound **5** was confirmed by comparing spectra to known NMR. ^{1}H NMR (500 MHz, $CDCl_3$) δ 9.45 (d, *J* = 7.8 Hz, 1H), 7.47 – 7.42 (m, 6H), 7.32 (dd, *J* = 8.5, 6.7 Hz, 6H), 7.25 (d, *J* = 7.2 Hz, 3H), 6.65 (dt, *J* = 15.7, 6.5 Hz, 1H), 6.03 – 5.97 (m, 1H), 2.38 (ddd, *J* = 9.7, 6.6, 2.3 Hz, 2H), 2.35 – 2.29 (m, 2H); ^{13}C NMR (126 MHz, $CDCl_3$) δ 193.75, 155.76, 144.58, 133.65, 129.54, 127.98, 126.80, 67.01, 31.73, 30.04.

Synthesis of (*E*)-3-hydroxy-*N*-methyl-*N*-phenyl-7-(tritylthio)hept-4-enamide, 7

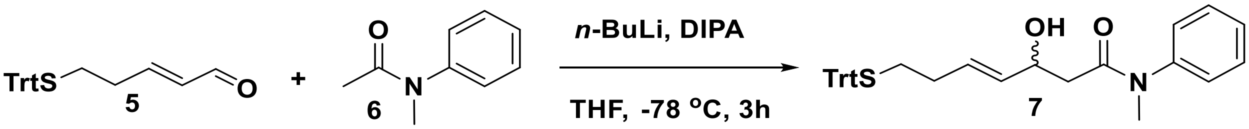

Under nitrogen, 50mL of THF (50mL) was poured into a round bottom flask and cooled on dry ice. Later, diisopropylamine (DIPA) (4.70 mL, 33.46 mmol, 4 equiv.) was added into the round bottom flask. In the next step, n-butyllithium (3.09 mL, 33.47 mmol, 4 equiv.) was added dropwise at -78°C and stirred for 1 hour. was added at -78°C and was allowed to stir for additional 1 hour. Lastly, compound#**5**, (*E*)-5-(tritylthio)pent-2-enal (3 g, 8.36 mmol, 1 equiv.) was added and the mixture was stirred for 1 hour under the same temperature. As a final step, the reaction was quenched using 25 mL of NH4Cl (40%) and concentrated in vacuo to remove THF. During the workup, dichloromethane was added to the aqueous mixture, and two phases were separated. Following the collection of the organic layer, dichloromethane was used twice to extract the aqueous layer before the organic layers were combined. The organic layer was washed with $NaHCO_3$ solution, brine, then dried over anhydrous Na_2SO_4, and concentrated in vacuo. The residue was purified with flash column chromatography on silica gel (ethyl acetate/ hexane, 1:9) to afford 3.2 g (75%) of product, (*E*)-3-hydroxy-*N*-methyl-*N*-phenyl-7-(tritylthio)hept-4-enamide, product#**7,** as a white solid. ^{1}H NMR (500 MHz, $CDCl_3$) δ 7.44 – 7.36 (m, 9H), 7.28 (dd, J = 9.4, 6.0 Hz, 6H), 7.24 – 7.19 (m, 3H), 7.17 (dd, J = 5.8, 3.5 Hz, 2H), 5.50 (dt, J = 15.8, 6.7 Hz, 1H), 5.30 (dd, J = 15.4, 5.9 Hz, 1H), 4.41 – 4.34 (m, 1H), 3.27 (s, 3H), 2.26 (dd, J = 16.3, 3.1 Hz, 1H), 2.21 (d, J = 8.3 Hz, 1H), 2.17 (t, J = 5.9 Hz, 2H), 2.09 – 2.00 (m, 2H). ^{13}C NMR (126 MHz, $CDCl_3$) δ 172.40, 144.91, 143.32, 132.21, 129.95, 129.59, 129.46, 128.13, 127.84, 127.24, 126.57, 68.96, 66.52, 40.30, 37.11, 31.45, 29.72.

Synthesis of (4*E*,4'*E*)-7,7'-disulfanediylbis(3-hydroxy-*N*-methyl-*N*-phenylhept-4-enamide), 8 (JRM-20)

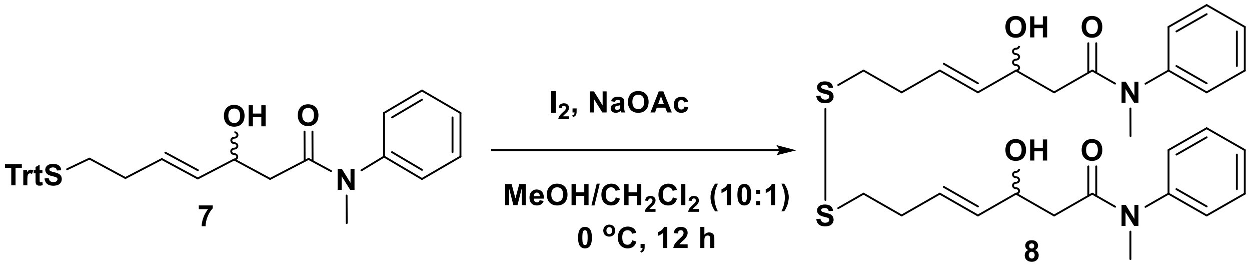

10:1 solution of CH_2Cl_2/MeOH (15 mL) solution was prepared in a round bottom flask under vacuum. Iodine (0.72 g, 2.83 mmol, 1.0 equiv.) and sodium acetate (0.46 g, 5.674 mmol, 2.0 equiv.) were dissolved into that under nitrogen atmosphere at 0 °C. Compound#**7**(1 g, 1.97 mmol, 1.0 equiv.) was dissolved in a 10:1 solution of CH_2Cl_2/MeOH (10 mL) and was added dropwise over 20 minutes to the first solution containing iodine and sodium acetate using an additional flask. This solution was then allowed to stir for additional 2 hours. The reaction was quenched by adding a saturated sodium thiosulfate ($Na_2S_2O_3$) solution until the mixture turned clear. Then, brine (5 mL) was added, and the layers were separated. The aqueous layer was extracted with dichloromethane (3 x 15 mL) and then with ethyl acetate (3 x 15 mL). The crude was purified with flash column chromatography on silica gel using hexane/ethyl acetate mobile phase to afford 0.8 g (76% yield) of product #**8** as a white solid. ^{1}H NMR (500 MHz, CDCl3) δ 7.46 – 7.41 (m, 2H), 7.39 – 7.34 (m, 1H), 7.20 – 7.15 (m, 2H), 5.67 – 5.59 (m, 1H), 5.42 (dd, *J* = 15.4, 6.1 Hz, 1H), 4.45 – 4.38 (m, 1H), 3.28 (s, 3H), 2.65 (dd, *J* = 11.4, 4.2 Hz, 2H), 2.37 – 2.28 (m, 2H), 2.27 – 2.17 (m, 2H). ^{13}C NMR (126 MHz, $CDCl_3$) δ 172.35, 143.29, 132.75, 129.98, 128.86, 128.17, 127.23, 68.95, 40.39, 38.11, 37.16, 31.92. HRMS (ESI+): Calculated (m/z) for $C_{28}H_{36}N_2O_4S_2$ $(M+H)^+$: 529.2189, Found 529.2202.

Synthesis of *S*-((*E*)-5-hydroxy-7-(methyl(phenyl)amino)-7-oxohept-3-en-1-yl) (*E*)-5-hydroxy-7-(methyl(phenyl)amino)-7-oxohept-3-ene-1-sulfinothioate, 9

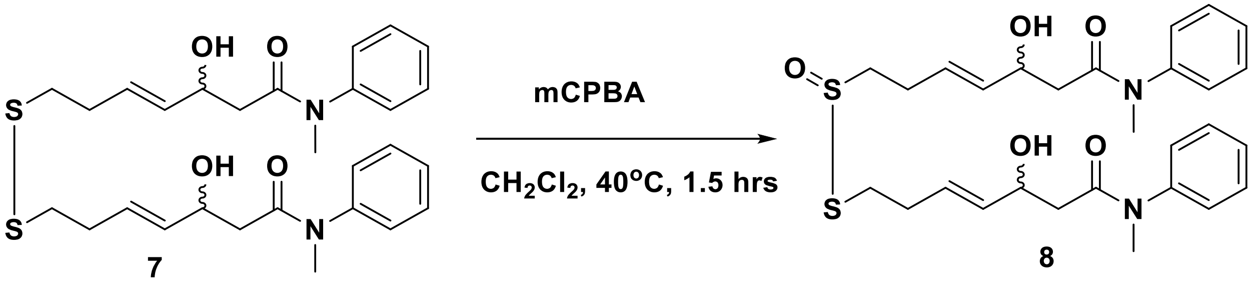

In a charged three-neck round bottom flask, a solution of **JRM-20** (0.7 g, 0.151 mmol, 1 equiv.) dissolved in 20 mL DCM was added under nitrogen condition. Then, mCPBA (26.1 mg, 0.151 mmol, 1.1 equiv.) dissolved in DCM was added to the round bottom flask dropwise. When the addition was done, the reaction was continued under reflux condition for 1.5 hours. The completion of reaction was confirmed by taking TLC at hexane/ethyl acetate, 7:3). The residue was purified

with flash column chromatography on silica gel hexane/ethyl acetate to afford 0.8 g (84% yield) of product, **9** as a brown solid. ^{1}H NMR (500 MHz, $CDCl_3$) δ 7.40 – 7.34 (m, 2H), 7.30 (td, *J* = 7.6, 3.2 Hz, 1H), 7.14 – 7.08 (m, 2H), 5.64 – 5.53 (m, 1H), 5.41 (ddd, *J* = 21.5, 10.8, 3.6 Hz, 1H), 4.41 – 4.34 (m, 1H), 3.21 (s, 3H), 3.07 (dd, *J* = 8.1, 5.6 Hz, 1H), 3.02 – 2.96 (m, 1H), 2.43 (dd, *J* = 15.5, 7.8 Hz, 1H), 2.40 – 2.32 (m, 1H), 2.20 (ddd, *J* = 16.3, 5.5, 2.6 Hz, 1H), 2.17 – 2.08 (m, 1H); ^{13}C NMR (126 MHz, $CDCl_3$) δ 172.26, 143.28, 143.22, 133.82, 133.69, 130.03, 128.25, 127.90, 127.21, 126.81, 68.76, 68.62, 55.16, 40.39, 40.27, 37.15, 33.49, 32.33, 26.01. HRMS (ESI+): Calculated (m/z) for $C_{28}H_{36}N_2O_5S_2$ $(M+H)^+$: 545.2138, Found 545.2133.

Synthesis of *S*-((*E*)-5-hydroxy-7-(methyl(phenyl)amino)-7-oxohept-3-en-1-yl) (*E*)-5-hydroxy-7-(methyl(phenyl)amino)-7-oxohept-3-ene-1-sulfonothioate, JRM-28

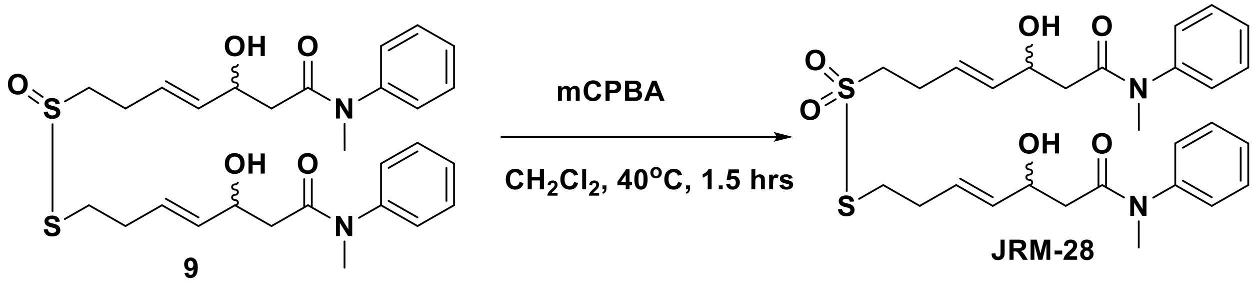

In a charged three-neck round bottom flask, a solution of compound **9** (70 mg, 0.151 mmol, 1 equiv.) dissolved in 20 mL DCM was added under nitrogen condition. Then, mCPBA (52 mg, 0.51 mmol, 2 equiv.) dissolved in DCM was added to the round bottom flask dropwise. When the addition was done, the reaction was continued under reflux condition for 1.5 hours. The completion of reaction was confirmed by taking TLC at hexane/ethyl acetate, 7:3). The residue was purified with flash column chromatography on silica gel hexane/ethyl acetate, 7:3) using triethyl amine (TEA) to afford 0.7 g (93% yield) of product, (4E,4'E)-7,7'-disulfanediylbis(3-hydroxy-1-(naphthalen-2-yl)hept-4-en-1-one), **JRM-28** as a light brown solid. 1H NMR (500 MHz, CDCl3) δ 7.49 – 7.43 (m, 2H), 7.42 – 7.37 (m, 1H), 7.20 (dd, *J* = 5.3, 4.4 Hz, 2H), 5.69 – 5.60 (m, 1H), 5.53 – 5.45 (m, 1H), 4.45 (dd, *J* = 8.9, 5.2 Hz, 1H), 3.34 – 3.30 (m, 1H), 3.30 (s, 3H), 3.13 (t, *J* = 7.3 Hz, 1H), 2.60 – 2.54 (m, 1H), 2.42 (dd, *J* = 14.3, 7.1 Hz, 1H), 2.33 – 2.27 (m, 1H), 2.24 – 2.18 (m, 1H); ^{13}C NMR (126 MHz, $CDCl_3$) δ 172.24, 172.19, 143.22, 143.20, 134.24, 134.15, 130.05, 128.28, 128.26, 127.21, 125.78, 68.57, 67.98, 61.74, 40.25, 40.21, 37.16, 32.36, 26.26, 25.62. HRMS (ESI+): Calculated (m/z) for $C_{28}H_{36}N_2O_6S_2$ $(M+H)^+$: 561.2087, Found 561.2090.

The justification of DTT treatment

JRM-28 generates two heterogeneous species (JRM-28a and JRM-28b) when a 19.3 µL solution of JRM-28 (10 mM) in DMSO was treated with 0.7 µL aqueous solution of DTT (350 mM) for 15 minutes at room temperature. DTT breaks the thiosulfonate bond in JRM-28 by nucleophilic attack (red curved arrows) and generates one thiol species (JRM-28a) and another sulfinic acid (JRM-28b) by a previously reported (1) route shown in figure 2A. The sulfinic acid species is more

thermodynamically stable than the thiol species (2). As a result, the reactive thiol species further undergoes a similar type of disulfide exchange reaction as DTT with JRM-28 to generate JRM-20 and JRM-28a (green curved arrows as shown in supplementary figure 16.) (3, 4). Due to the symmetrical structure of precursor molecule JRM-20, DTT treatment generates only JRM-20b. The mass spectrum of the sample containing JRM-28 and DTT confirms the existence of JRM-28a (trace), JRM-20 (trace), and JRM-28 (predominant, base peak at 561) in positive ion mode (Figure 2C), whereas the negative ion mode predominantly confirmed the generation of JRM-28a (Figure 2D). The mass spectrum for JRM-20 with DDT displayed the base peak at 266 for JRM-28b (Figure 2D).

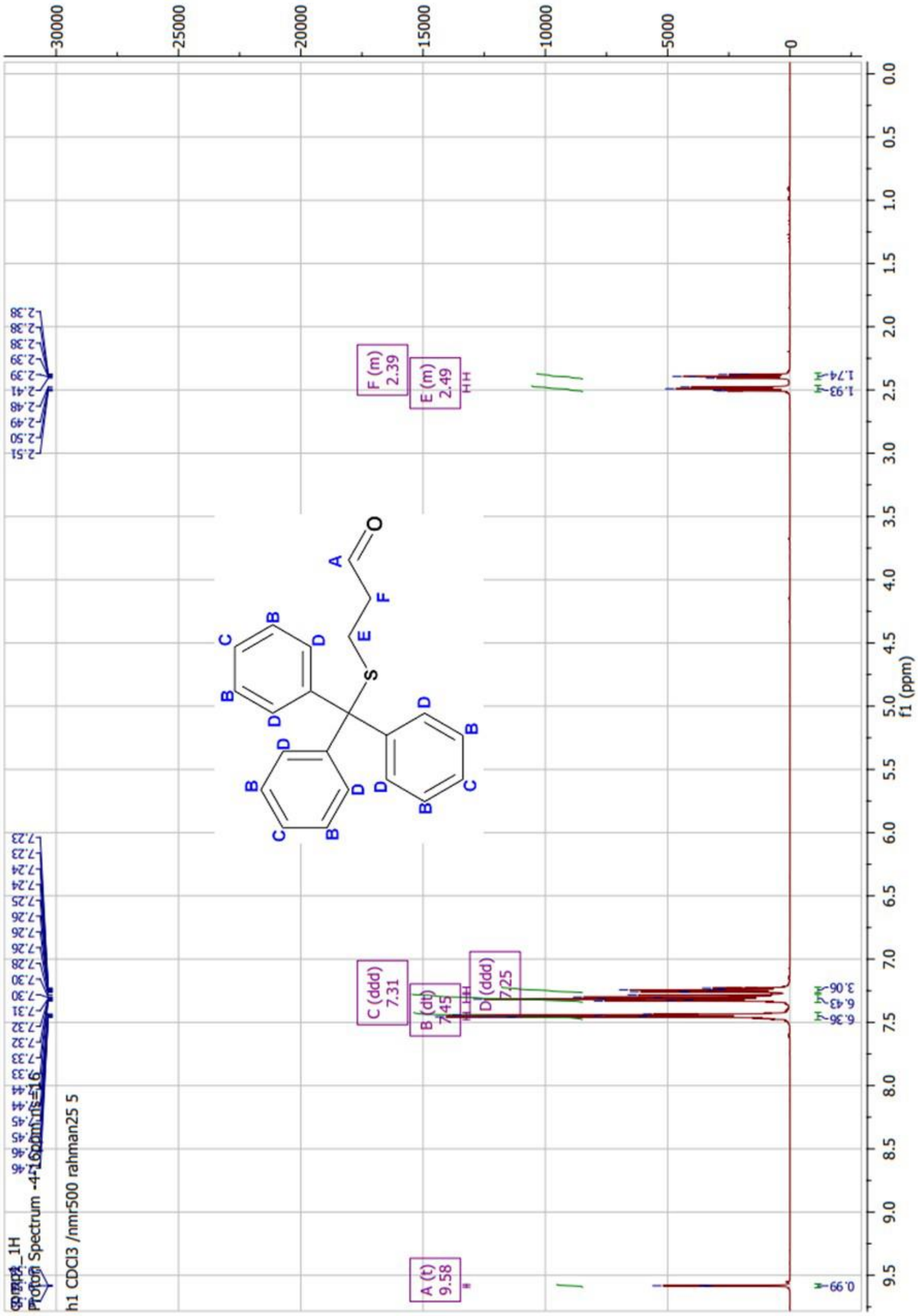

Supplementary Figure S1: ^{1}H-NMR of 3-(Tritylthio)propanal (Compound #3 mentioned in Figure 1A).

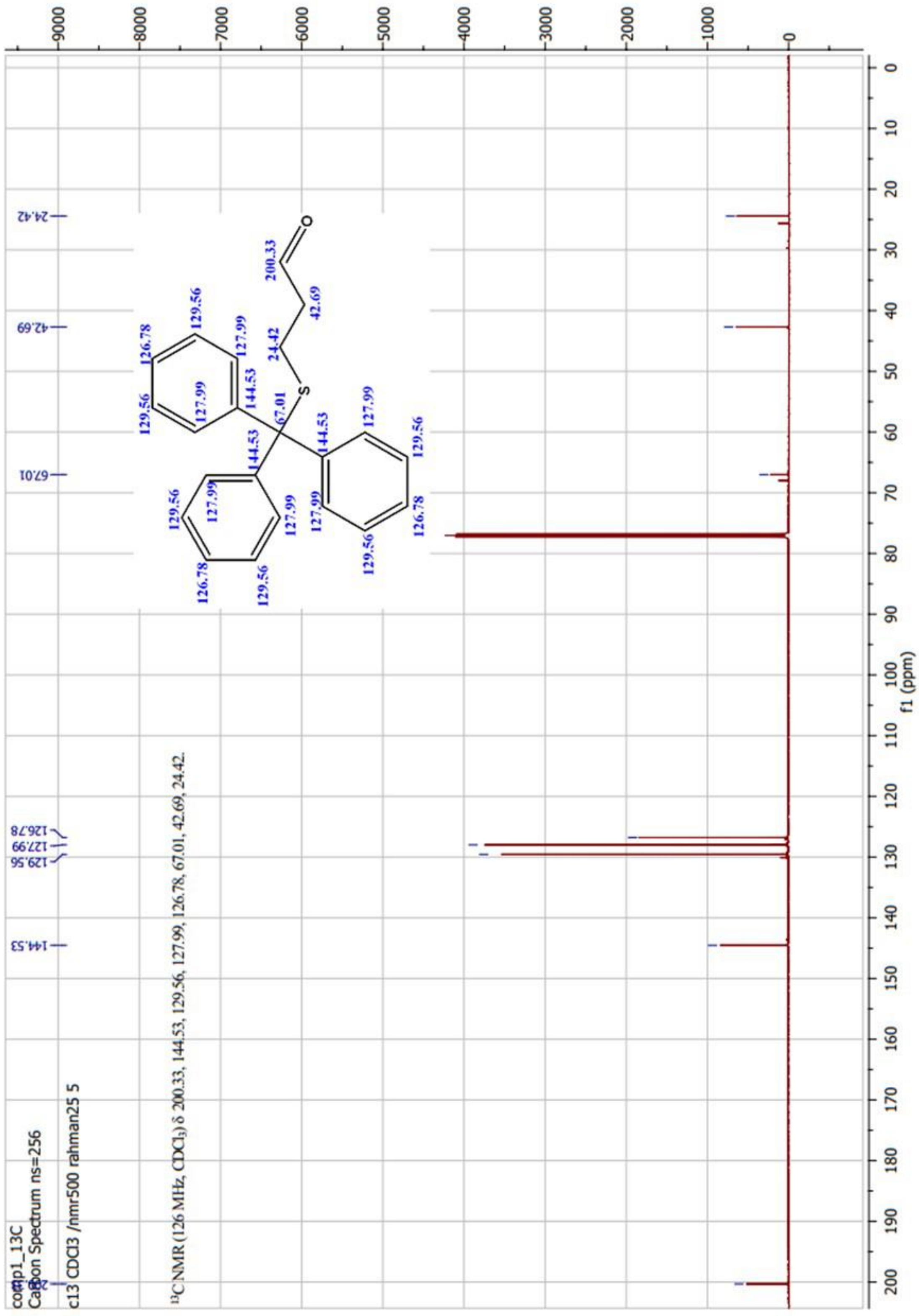

Supplementary Figure S2: ^{13}C-NMR of 3-(Tritylthio)propanal (Compound #3 mentioned in Figure1A)

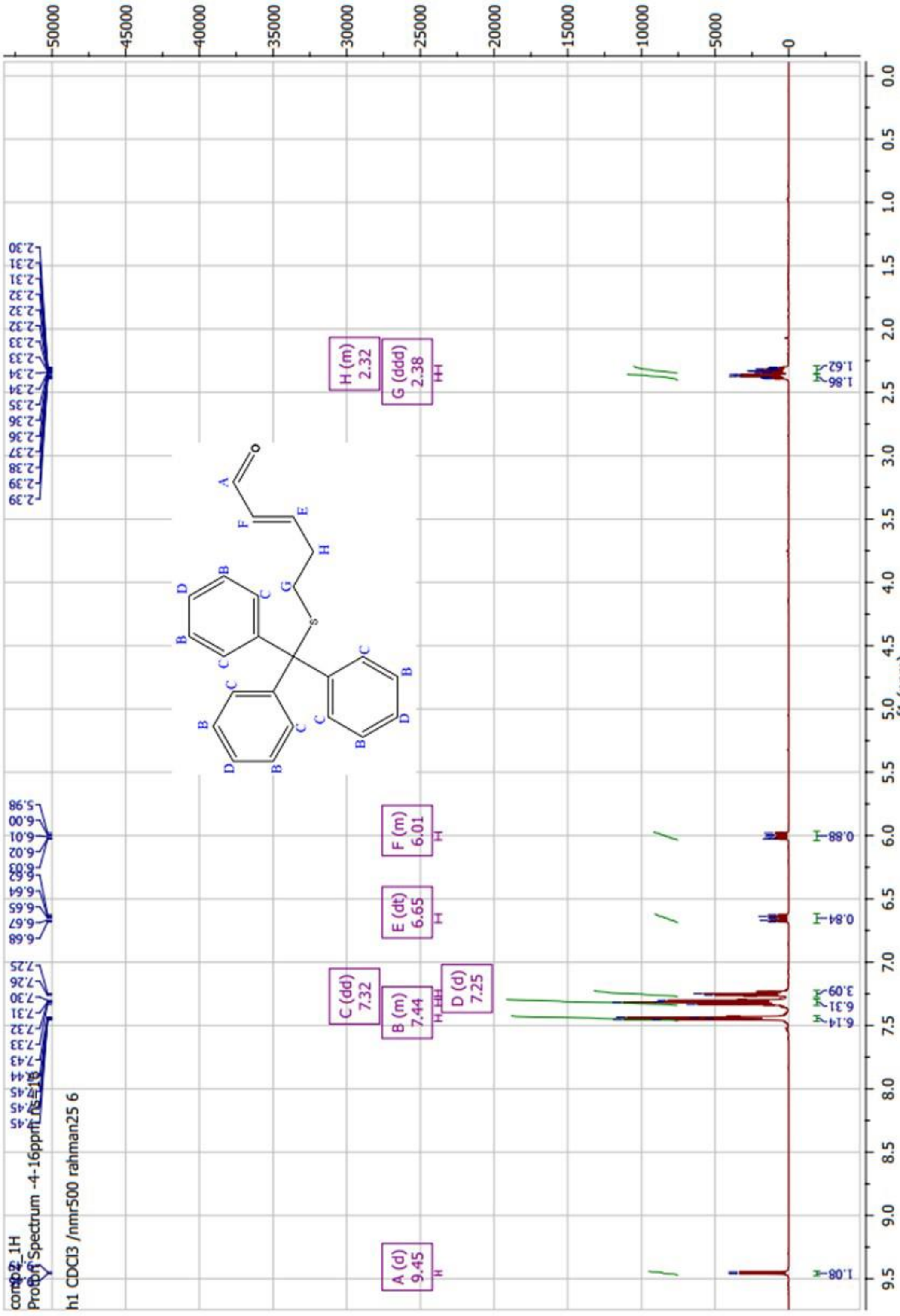

Supplementary Figure S3: ^{1}H-NMR of (*E*)-5-(tritylthio)pent-2-enal (Compound #5 mentioned in Figure1A)

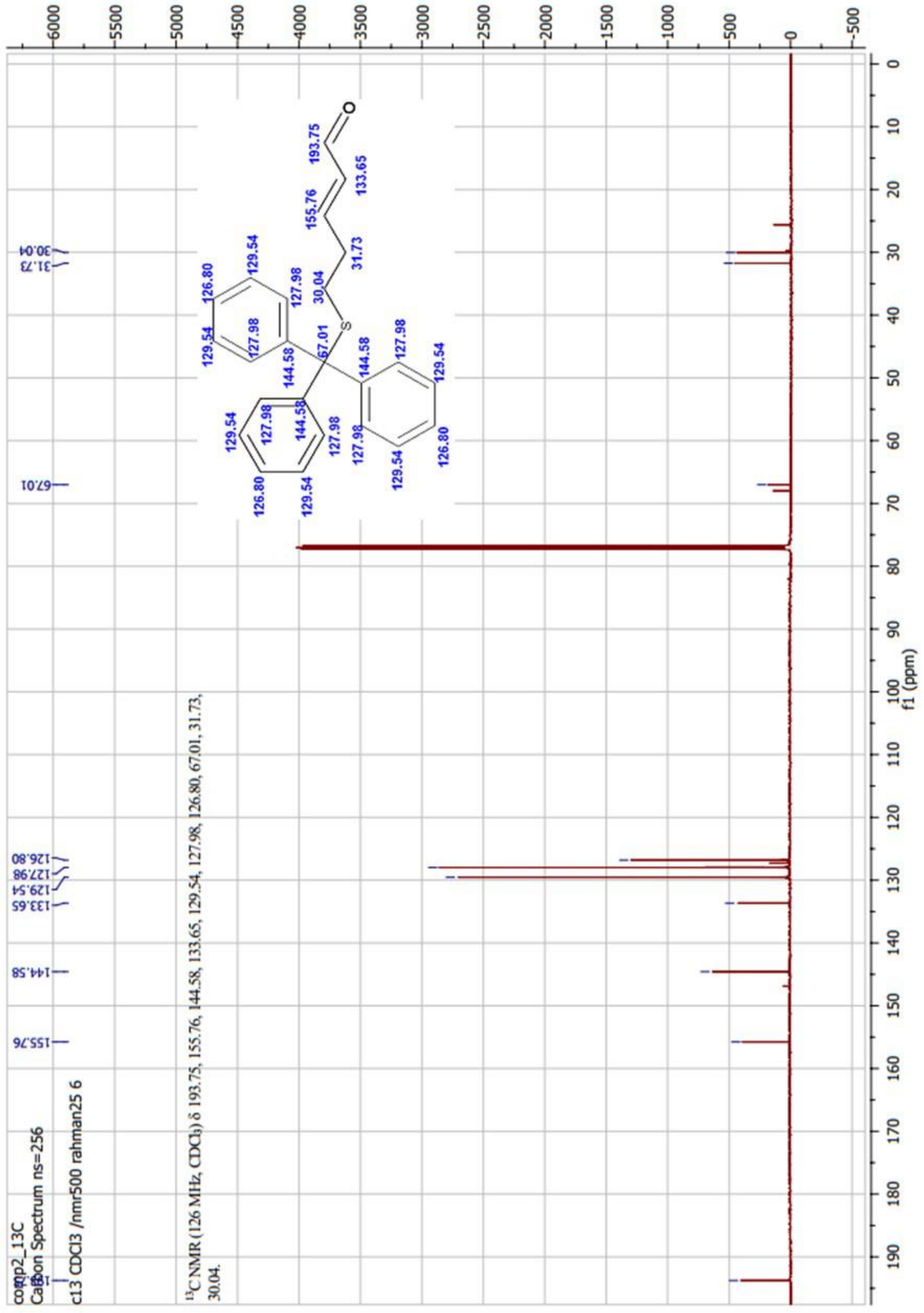

Supplementary Figure S4: ^{13}C-NMR of (*E*)-5-(tritylthio)pent-2-enal (Compound #5 mentioned in Figure1A)

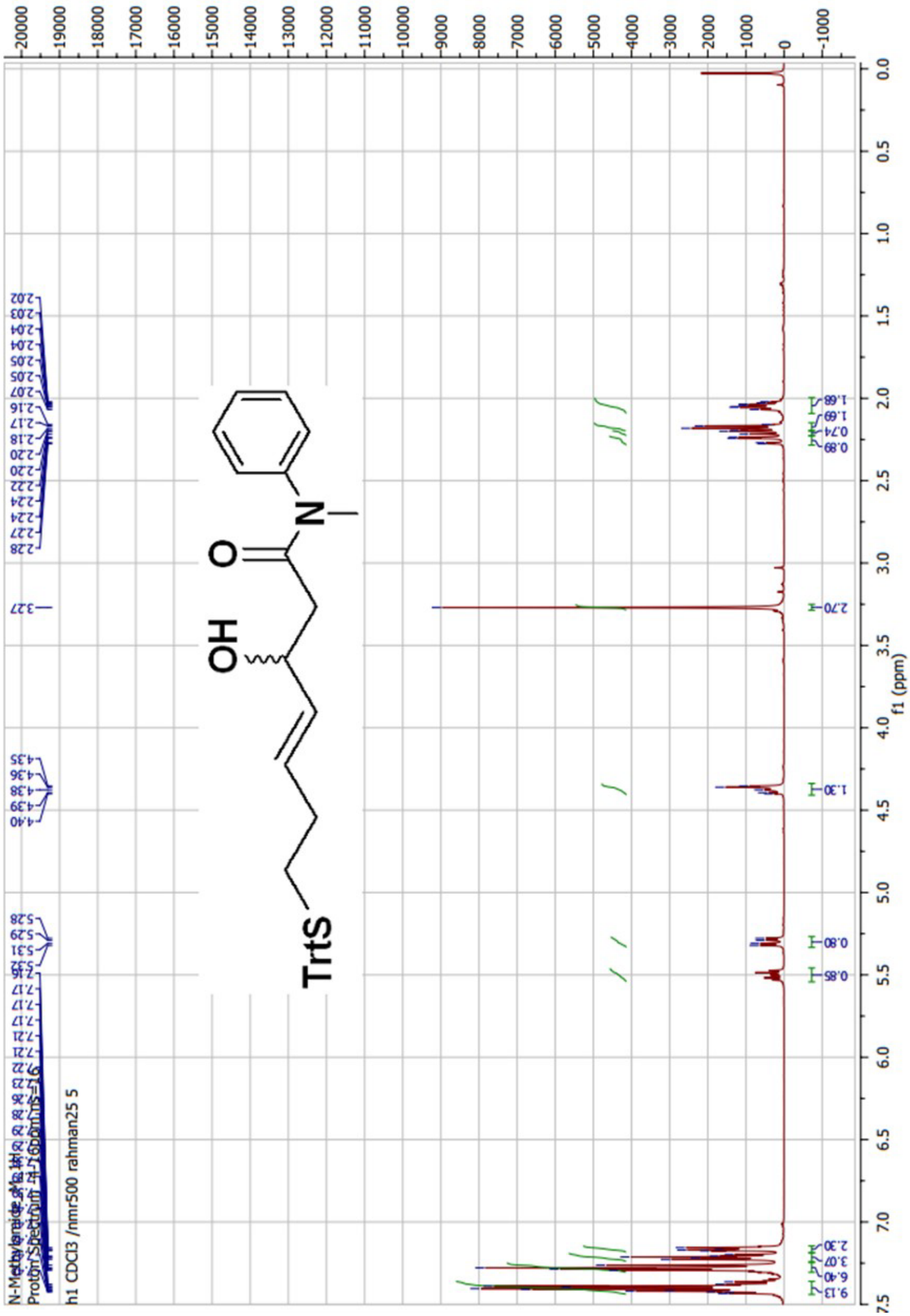

Supplementary Figure S5: ^{1}H-NMR of (*E*)-3-hydroxy-*N*-methyl-*N*-phenyl-7-(tritylthio)hept-4-enamide (Compound #7 mentioned in Figure1A)

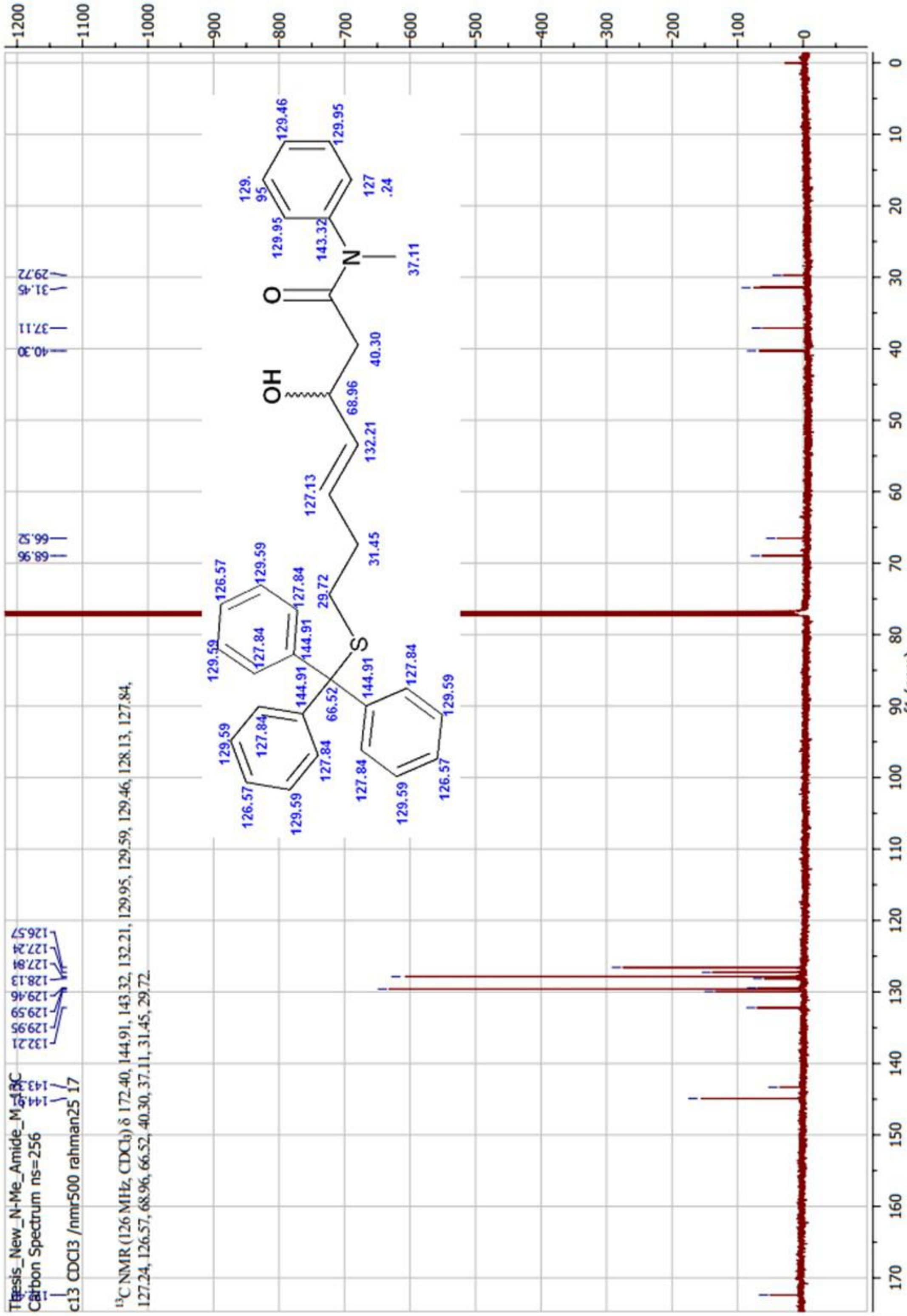

Supplementary Figure S6: ^{13}C-NMR of (*E*)-3-hydroxy-*N*-methyl-*N*-phenyl-7-(tritylthio)hept-4-enamide (Compound #7 mentioned in Figure1A)

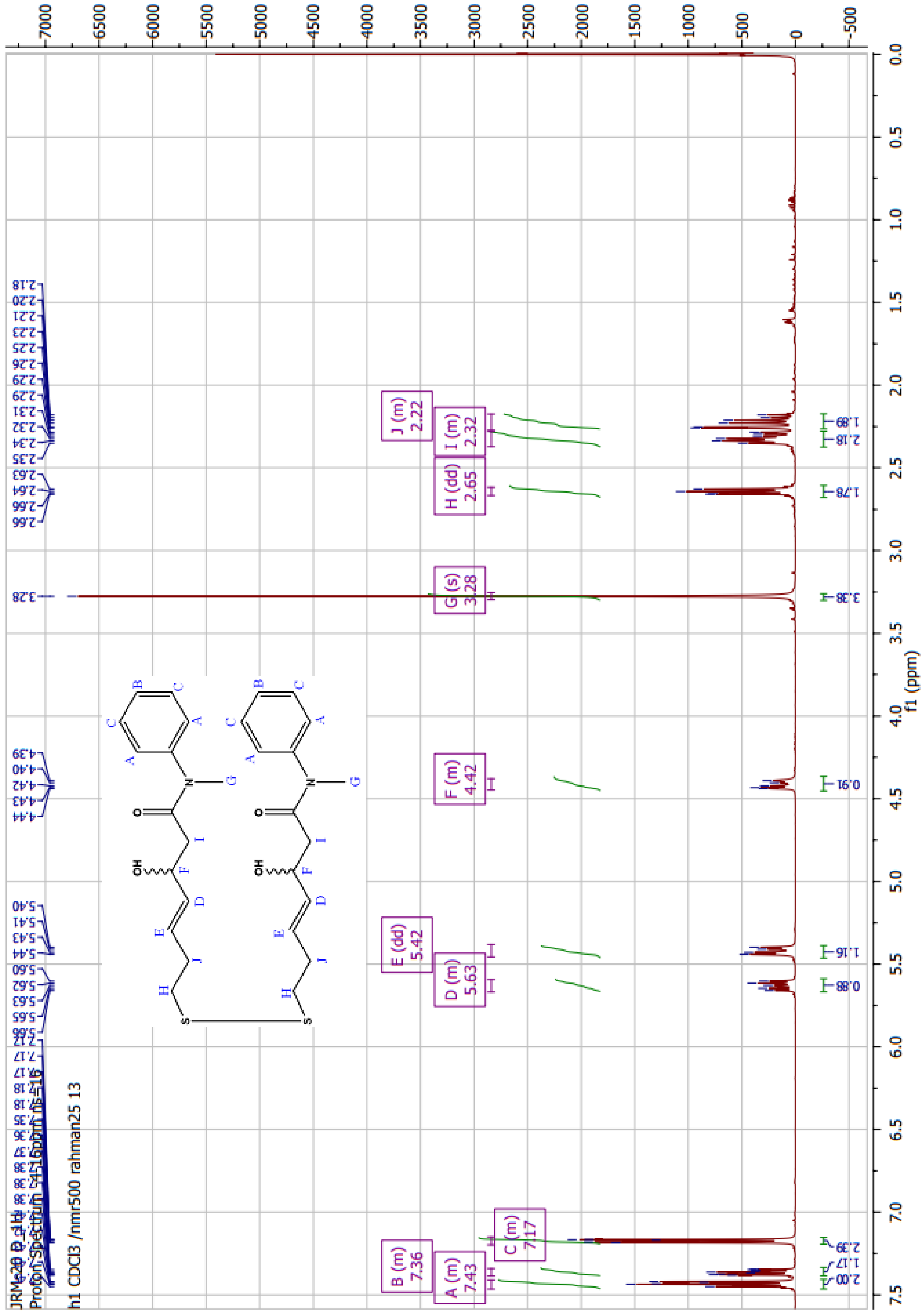

Supplementary Figure S7: ^{1}H-NMR of (4*E*,4'*E*)-7,7'-disulfanediylbis(3-hydroxy-*N*-methyl-*N*-phenylhept-4-enamide) (Compound #8 mentioned in Figure1A)

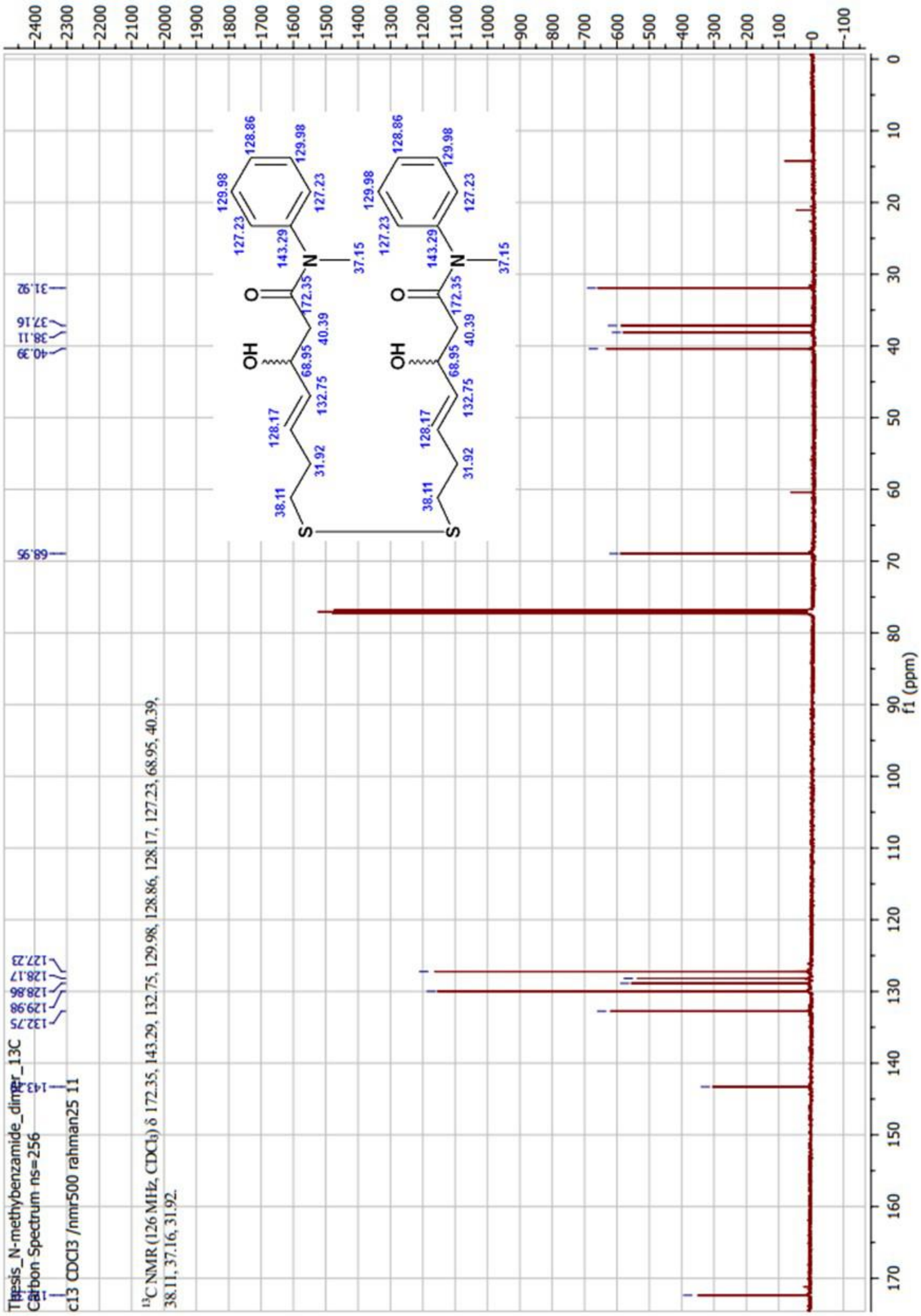

Supplementary Figure S8: ^{13}C-NMR of (4*E*,4'*E*)-7,7'-disulfanediylbis(3-hydroxy-*N*-methyl-*N*-phenylhept-4-enamide) (Compound #8 mentioned in Figure1A)

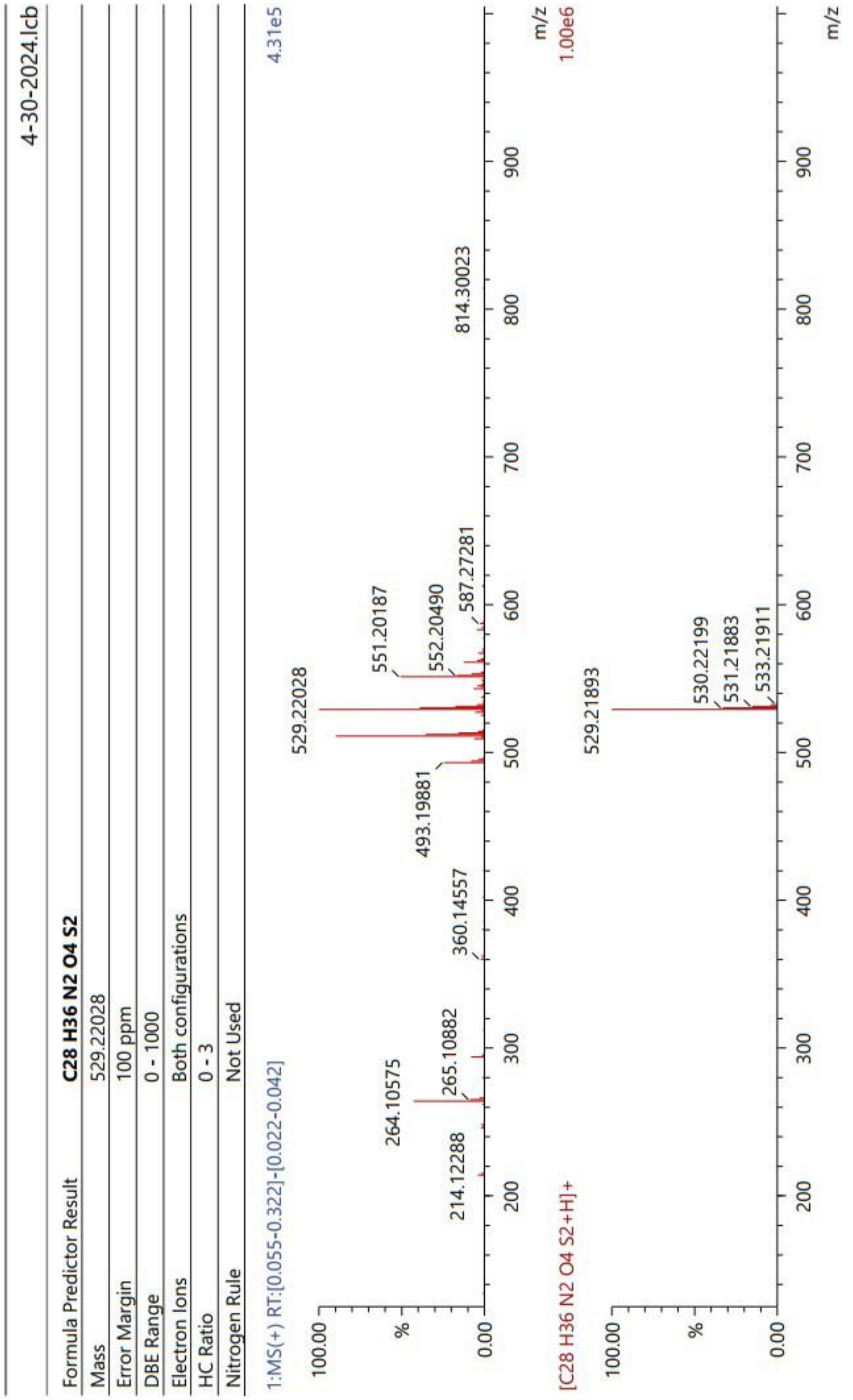

Supplementary Figure S9: HRMS of (4*E*,4'*E*)-7,7'-disulfanediylbis(3-hydroxy-*N*-methyl-*N*-phenylhept-4-enamide) (Compound #8 mentioned in Figure1A)

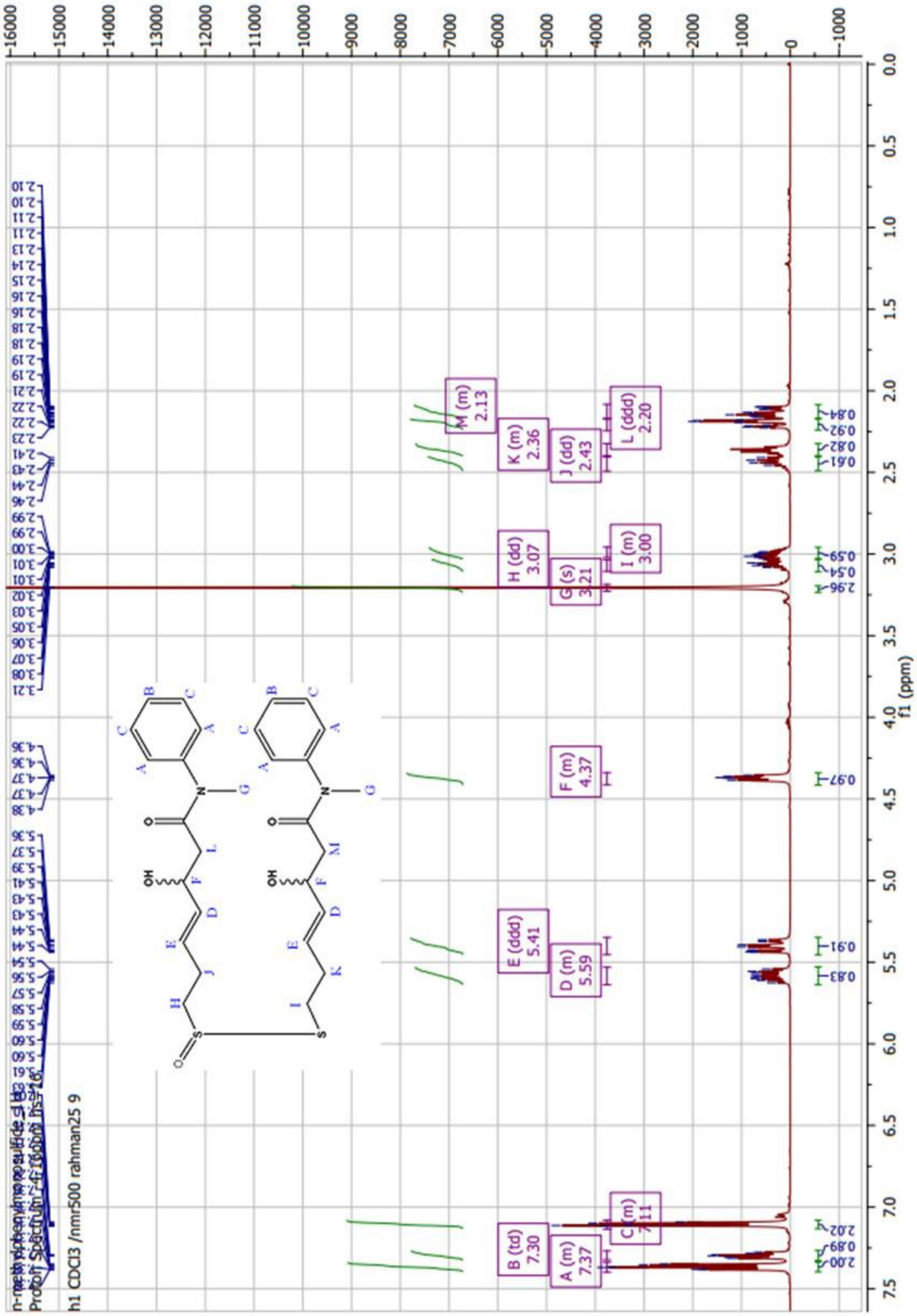

Supplementary Figure S10: ^{1}H-NMR of *S*-((*E*)-5-hydroxy-7-(methyl(phenyl)amino)-7-oxohept-3-en-1-yl) (*E*)-5-hydroxy-7-(methyl(phenyl)amino)-7-oxohept-3-ene-1-sulfinothioate (Compound #9 mentioned in Figure1A)

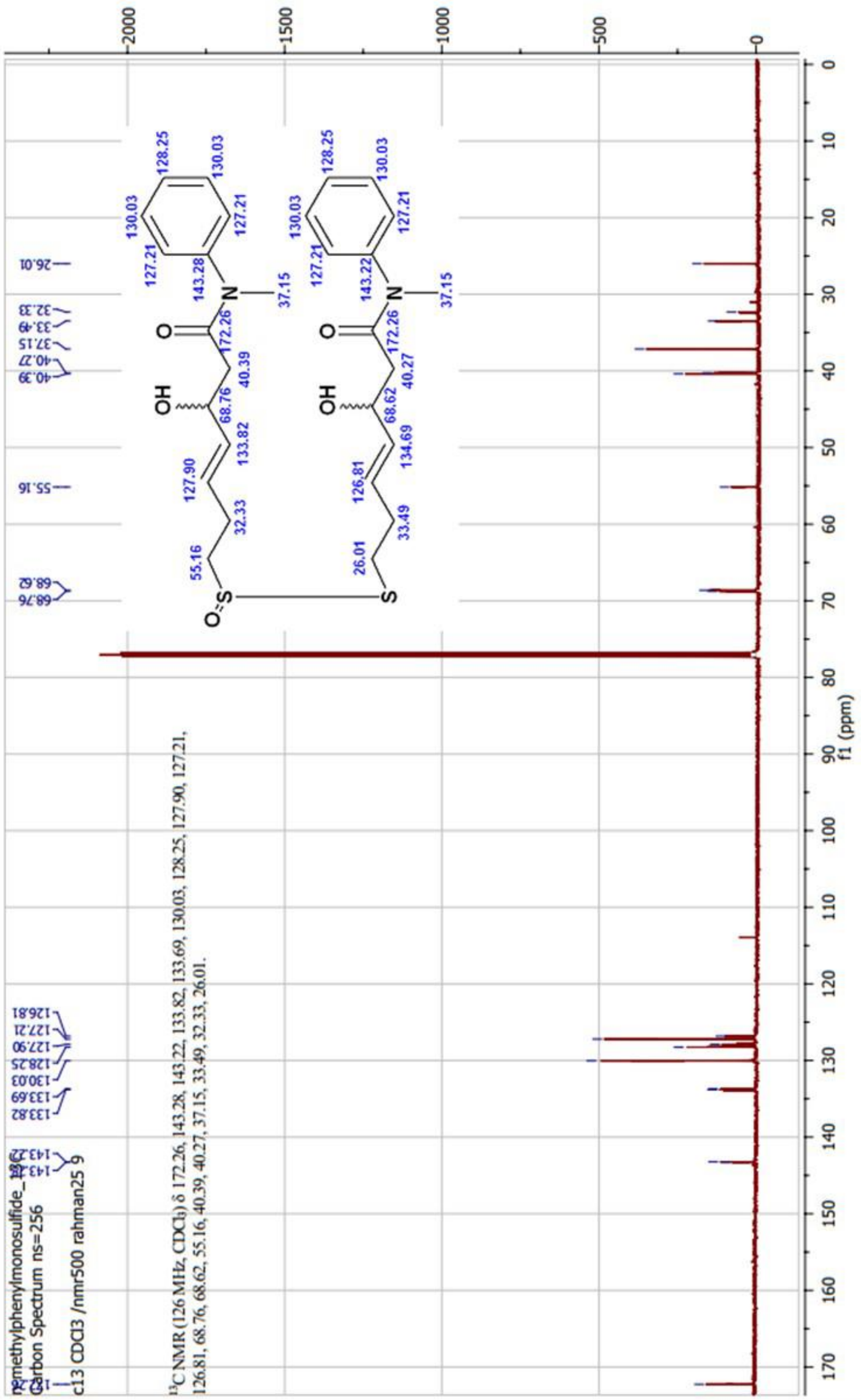

Supplementary Figure S11: ^{13}C-NMR of *S*-((*E*)-5-hydroxy-7-(methyl(phenyl)amino)-7-oxohept-3-en-1-yl) (*E*)-5-hydroxy-7-(methyl(phenyl)amino)-7-oxohept-3-ene-1-sulfinothioate (Compound #9 mentioned in Figure1A)

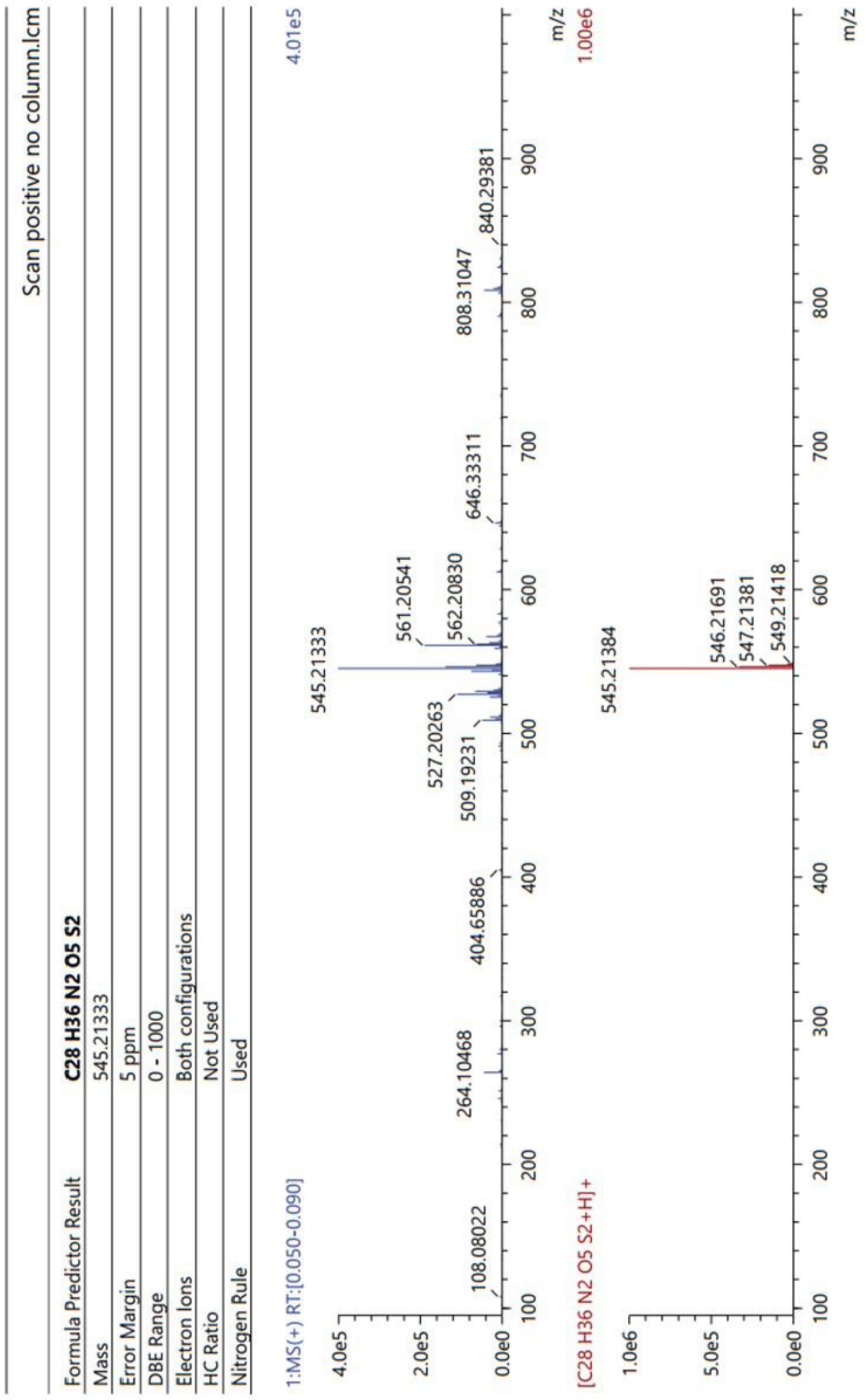

Supplementary Figure S12: HRMS of *S*-((*E*)-5-hydroxy-7-(methyl(phenyl)amino)-7-oxohept-3-en-1-yl) (*E*)-5-hydroxy-7-(methyl(phenyl)amino)-7-oxohept-3-ene-1-sulfinothioate (Compound #9 mentioned in Figure1A)

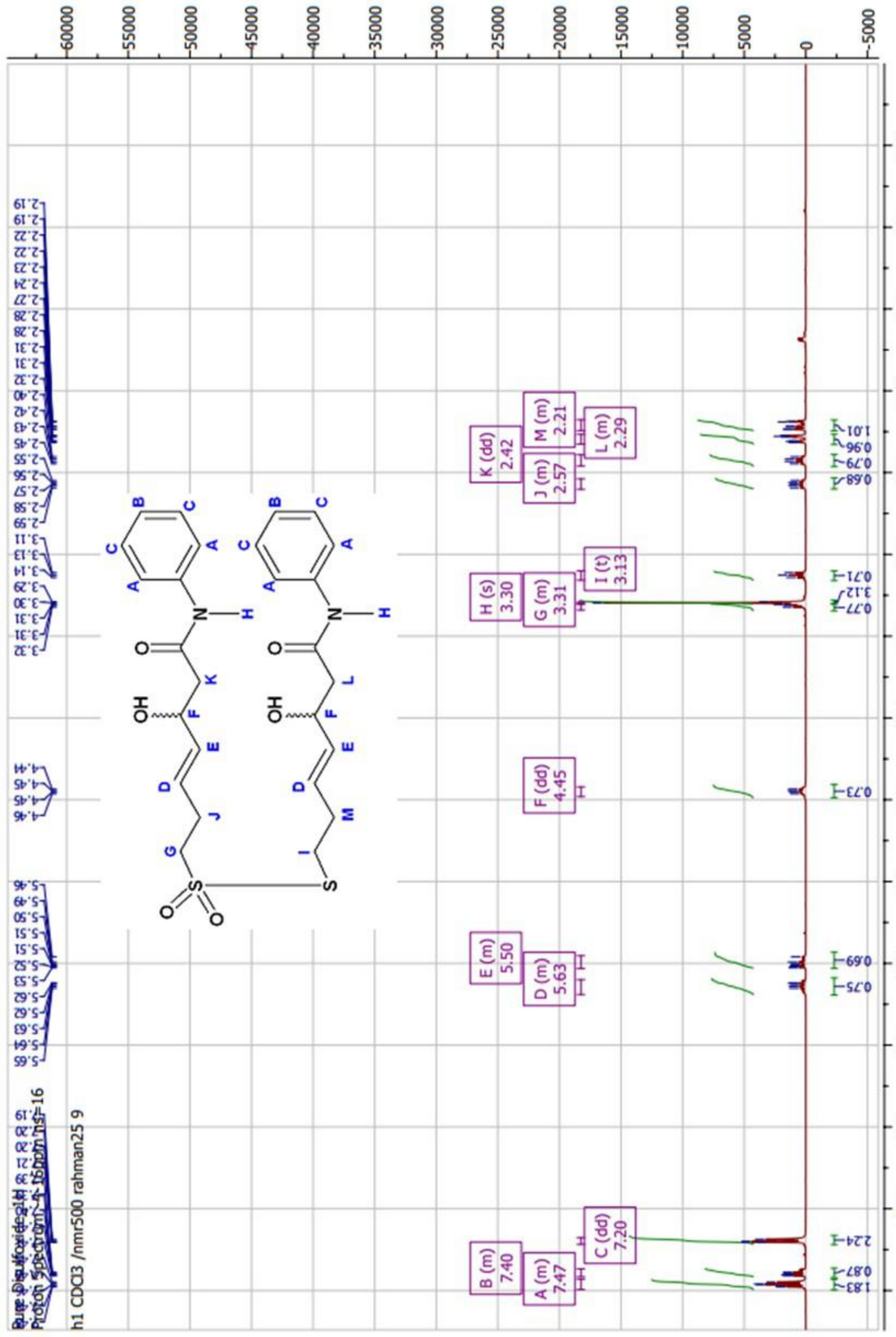

Supplementary Figure S13: ^{1}H-NMR of *S*-((*E*)-5-hydroxy-7-(methyl(phenyl)amino)-7-oxohept-3-en-1-yl) (*E*)-5-hydroxy-7-(methyl(phenyl)amino)-7-oxohept-3-ene-1-sulfonothioate (JRM-28 mentioned in Figure1A)

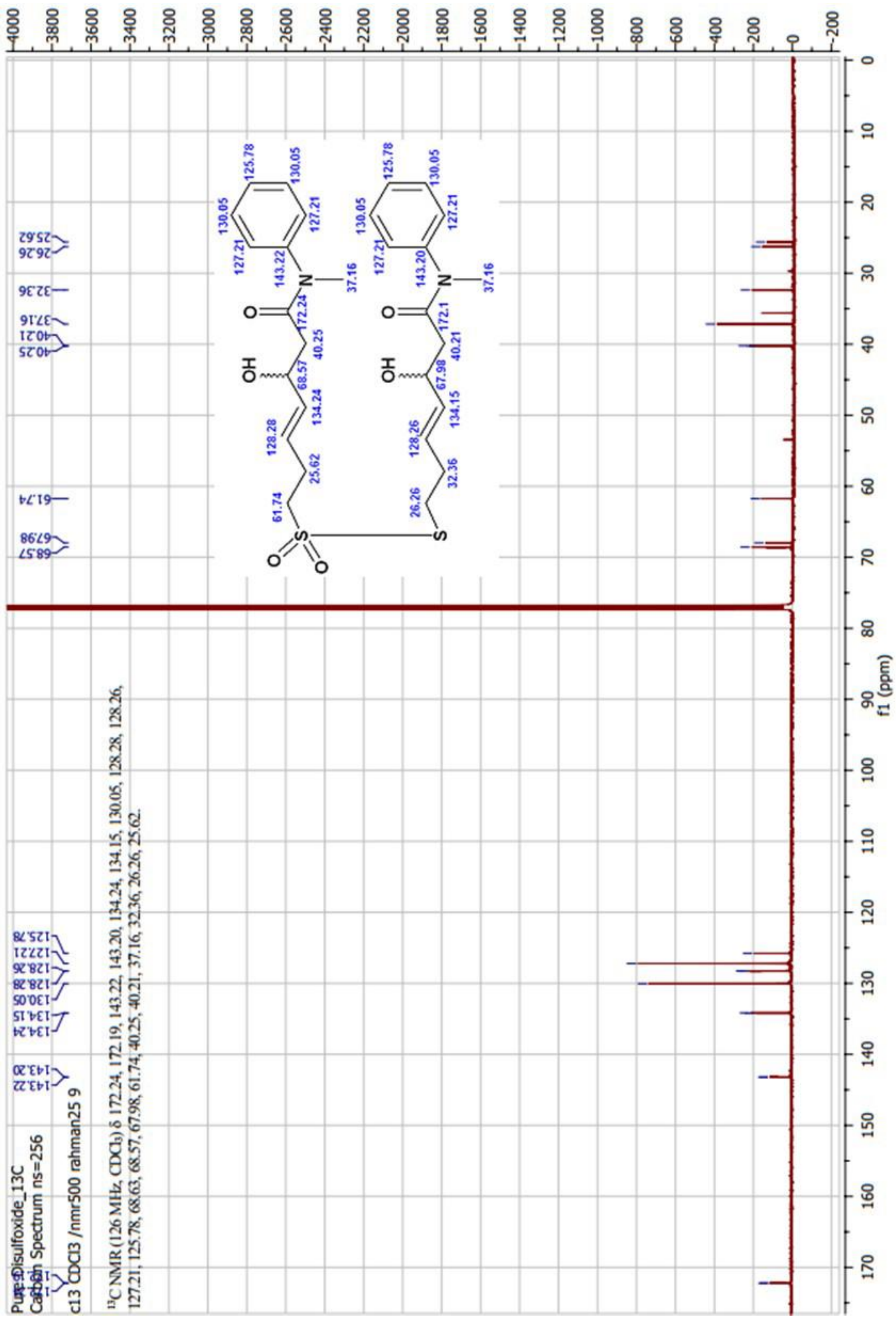

Supplementary Figure S14: ^{13}C-NMR of *S*-((*E*)-5-hydroxy-7-(methyl(phenyl)amino)-7-oxohept-3-en-1-yl) (*E*)-5-hydroxy-7-(methyl(phenyl)amino)-7-oxohept-3-ene-1-sulfonothioate (JRM-28 mentioned in Figure1A)

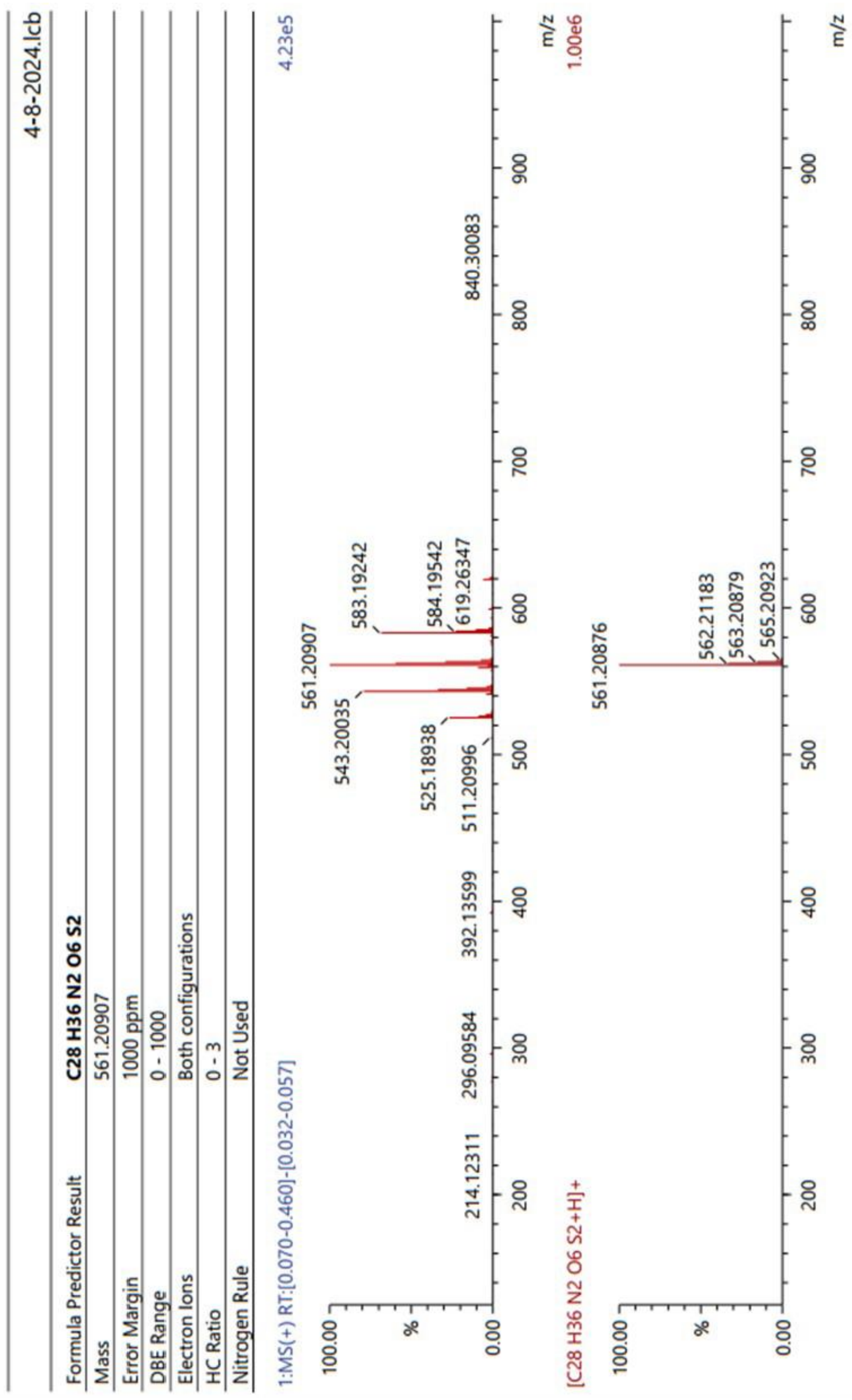

Supplementary Figure S15: HRMS of *S*-((*E*)-5-hydroxy-7-(methyl(phenyl)amino)-7-oxohept-3-en-1-yl) (*E*)-5-hydroxy-7-(methyl(phenyl)amino)-7-oxohept-3-ene-1-sulfonothioate (JRM-28 mentioned in Figure1A)

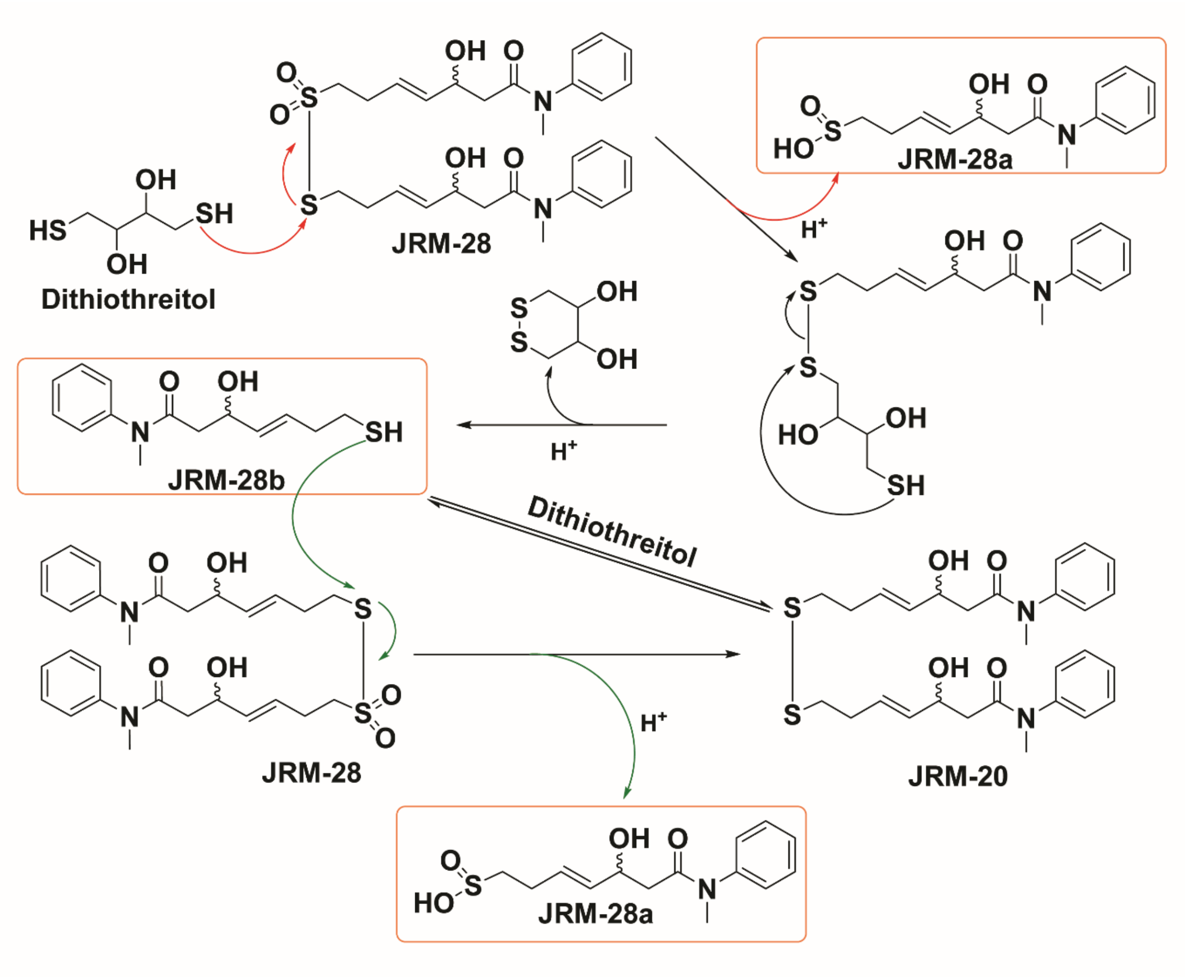

Supplementary Figure S16: Predicted schema of DTT-mediated reduction of JRM-28

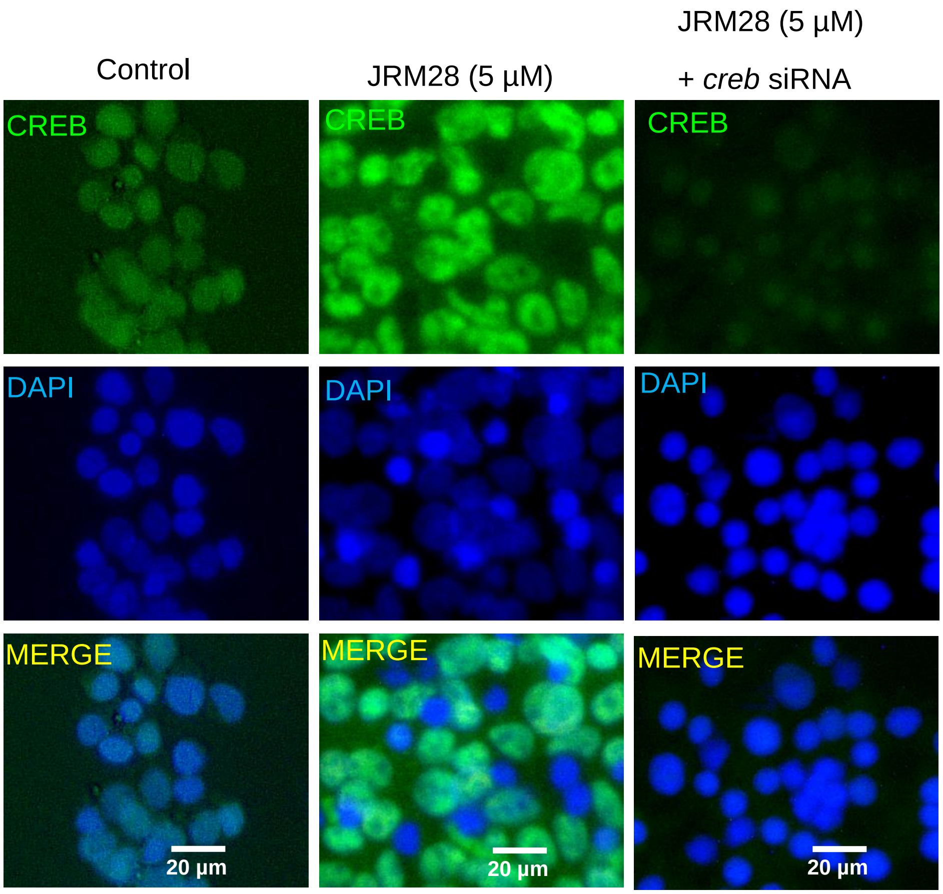

Supplementary Figure S17: Efficacy of creb siRNA. (A) SHSY5Y cells were treated with 0.1 µg of creb siRNA as described under method section. After 24 hrs, cells were treated with JRM28 and after another 24 hrs cells were fixed and stained with CREB antibody. Nuclei were stained with DAPI.

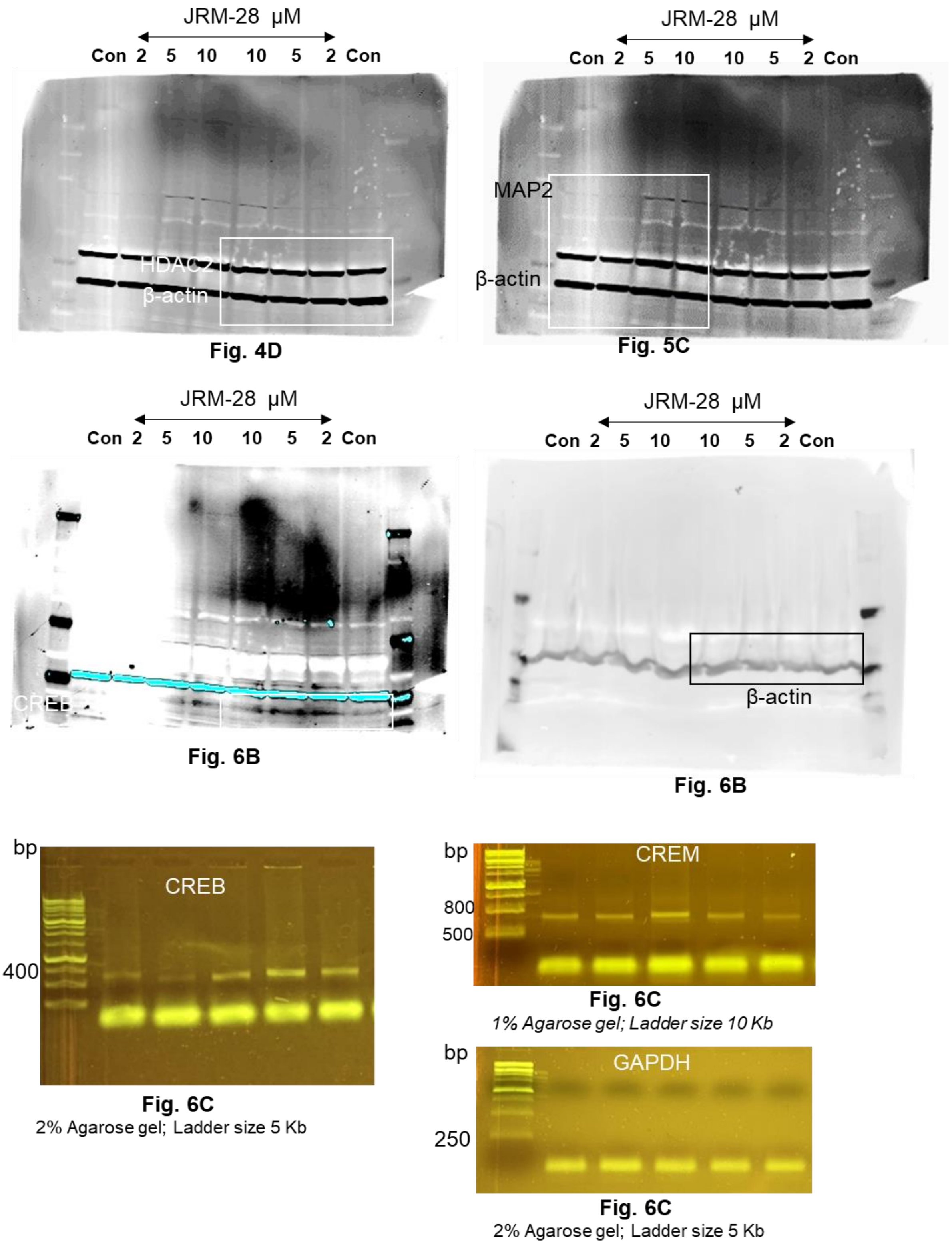

Supplementary Figure S18: Raw western blot and RT-PCR images used in the manuscript.

References:

1. Singh, R., andWhitesides, G. M. (1995) Reagents for rapid reduction of disulfide bonds in proteins In Techniques in Protein Chemistry, Crabb JW, ed. Academic Press, 259-266
2. Li, X., Gluth, A., Zhang, T., andQian, W. J. (2023) Thiol redox proteomics: Characterization of thiol-based post-translational modifications Proteomics **23**, e2200194 10.1002/pmic.202200194
3. Fernandes, P. A., andRamos, M. J. (2004) Theoretical insights into the mechanism for thiol/disulfide exchange Chemistry **10**, 257-266 10.1002/chem.200305343
4. Ge, C., Wang, H., Zhang, B., Yao, J., Li, X., Feng, W. *et al.* (2015) A thiol-thiosulfonate reaction providing a novel strategy for turn-on thiol sensing Chem Commun (Camb) **51**, 14913-14916 10.1039/c5cc05390k
